# Rational Design of Highly Potent and Slow-Binding Cytochrome *bc*_1_ Inhibitor as Fungicide by Computational Substitution Optimization

**DOI:** 10.1038/srep13471

**Published:** 2015-08-26

**Authors:** Ge-Fei Hao, Sheng-Gang Yang, Wei Huang, Le Wang, Yan-Qing Shen, Wen-Long Tu, Hui Li, Li-Shar Huang, Jia-Wei Wu, Edward A. Berry, Guang-Fu Yang

**Affiliations:** 1 Key Laboratory of Pesticide & Chemical Biology, Ministry of Education, College of Chemistry, Central China Normal University, Wuhan 430079, P.R. China; 2 Collaborative Innovation Center of Chemical Science and Engineering, Tianjing 300072, P.R. China; 3 MOE Key Laboratory of Protein Sciences, Tsinghua-Peking Center for Life Sciences, School of Life Sciences, Tsinghua University, Beijing 100084, P.R. China; 4 Department of Biochemistry and Molecular Biology, SUNY Upstate Medical University, Syracuse, NY 13210, USA

## Abstract

Hit to lead (H2L) optimization is a key step for drug and agrochemical discovery. A critical challenge for H2L optimization is the low efficiency due to the lack of predictive method with high accuracy. We described a new computational method called Computational Substitution Optimization (CSO) that has allowed us to rapidly identify compounds with cytochrome *bc*_1_ complex inhibitory activity in the nanomolar and subnanomolar range. The comprehensively optimized candidate has proved to be a slow binding inhibitor of *bc*_1_ complex, ~73-fold more potent (*K*_i_ = 4.1 nM) than the best commercial fungicide azoxystrobin (AZ; *K*_i_ = 297.6 nM) and shows excellent *in vivo* fungicidal activity against downy mildew and powdery mildew disease. The excellent correlation between experimental and calculated binding free-energy shifts together with further crystallographic analysis confirmed the prediction accuracy of CSO method. To the best of our knowledge, CSO is a new computational approach to substitution-scanning mutagenesis of ligand and could be used as a general strategy of H2L optimisation in drug and agrochemical design.

Agrochemicals play a significant role in modern agriculture by protecting crops against yield loss. Due to the increasing demand for food supply and the explosive development of resistance, the discovery and development of new agrochemicals are continuously demanded^1^. However, agrochemical discovery remains a vital challenge^2^: the research and development (R&D) costs have been rising from U.S. $152 million in 1995 to $256 million in 2005 and the number of compounds needs to be synthesized have been rising from 52500 in 1995 to 140000 in 2005 to bring a new agrochemical to market. A new agrochemical is invented initially by the discovery of a bioactive lead compound. Thus, lead generation is a crucial step for the agrochemical discovery^3^, which requires at least two processes: hit identification and Hit-to-Lead (H2L) optimization^4^. Over the past decade, introduction of new technologies, such as high-throughput screening (HTS) and fragment-based design, have made hit identification become more efficient^5^,^6^. But, traditional H2L optimization is still carried out through a large number of analogues’ syntheses, which lead to a low efficient and resource-intensive research^7^. Although some techniques, such as quantitative structure-activity relationship (QSAR)^8^ and molecular dynamics (MD) simulations^9^,^10^, have been developed to help understand the structure-activity relationship for H2L optimization, they still have some drawbacks, such as dependent of sufficient biological data, highly time-consuming, and low prediction accuracy^11^. Therefore, there is urgent demand in developing an effective tool enabling to eliminate undesirable lead compound classes/types early during H2L optimization process, prior to expensive and extensive experimental work^12^. Having this challenge in mind, we developed a new computational protocol called Computational Substitution Optimization (CSO) to rapidly assess how replacing hydrogen atoms with new substituent into a “hit” scaffold could improve binding free energies to their targets. To showcase this method, we applied CSO in the H2L optimization of cytochrome *bc*_1_ complex inhibitors to discover antifungal lead.

The cytochrome *bc*_1_ complex (EC 1.10.2.2, *bc*_1_) has been chosen as a model for the present study, because of its crucial role in the life cycle. The *bc*_1_ complex is an essential respiratory enzyme complex present in the inner mitochondrial membrane of eukaryotic organisms. It has been identified as a promising target for new drugs and agrochemicals^13^. For example, malaria, as a devastating tropical disease, remains a major threat to over 40% of the world’s population and approximately 660,000 deaths in 2010^14^. Atovaquone is a major drug for prophylaxis and treatment of uncomplicated malaria patient^15^ and as alternative therapy in case of resistances against chloroquine or artemisin-based therapies^16^. But with a limited number of antimalarial drugs available, the emergence of multidrug-resistant *Plasmodium falciparum* malaria threatens the health of individual patients as well as eradication strategies for the disease^17^. In addition, some *bc*_1_ complex inhibitors have been introduced into the agrochemical market to provide effective control of important fungal diseases threatening global food security^18^. Azoxystrobin (AZ), as a representative inhibitor targeting *bc*_1_, is the biggest using fungicide in the whole word with a global annual sales of over USD1.4 billion^19^. However, the increasing number of resistance is also a big problem that AZ has to face^19^. Therefore, there is a wide research interest on the discovery of cytochrome *bc*_1_ complex inhibitor today, which could be used as a specific probe for novel functional study and as a new lead compound for future drug and agrochemical discovery^20^.

In this study, the application of CSO has led to a series of new *bc*_1_ inhibitors with nanomolar to subnanomolar potency. Excellent agreements between the predicted and experimental binding free energies were obtained, indicating the predictive capacity of CSO. Further, assays showed that compound **18** was the lead candidate from the new series of *bc*_1_ complex inhibitors because of its high potency (*K*_i_ = 4.10 nM which is up to 73-fold higher than that of AZ), outstanding physiochemical properties, and excellent *in vivo* bioactivity. In order to investigate the underlying mechanisms responsible for the excellent *in vivo* bioactivity, the kinetic effects on cytochrome *bc*_1_ complex were studied and exhibited slow-binding characteristics, which is different from the classical fast-binding of AZ indicating a turning point for *bc*_1_ complex inhibitor discovery. Most importantly, the determined crystal structure of lead compound bound to *bc*_1_ complex at 3.23 Å provided a solid molecular basis for the prediction accuracy of CSO method. This work demonstrated that CSO method can significantly improve the efficiency of H2L optimization process and could be used as a general strategy for drug and agrochemical discovery.

## Results

### H2L Optimization through CSO

**Figure 1:**
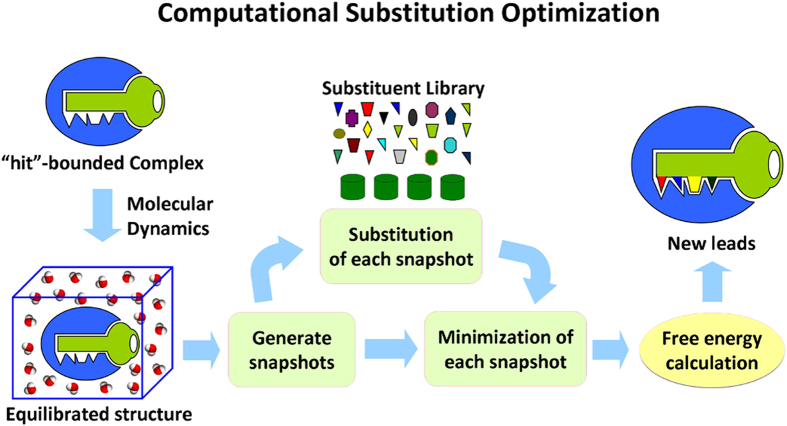
Diagrammatic representation of the workflow of CSO. The CSO protocol was designed to automatically perform computational substitution, energy minimization, and binding affinity evaluation: (1) The MD simulation of protein with original “hit” ligand was performed. (2) In order to obtain a representative ensemble of the binding structures, snapshots were collected from the MD trajectory at regular intervals and minimized. (3) The “Hit” ligand in each snapshot collected from the MD trajectory was mutated to new ligand with substitutions using the library of substitution and minimized. Each substitution group was set to link with the specific site of “Hit” compounds. (4) ΔΔ*G* was calculated and the final binding free energy change is the average of the calculated values associated with each snapshot. New leads were finally determined according to the order of the binding free-energy shifts.

The workflow of the computational protocol used in this study is shown in Fig. 1. This new protocol is a combination of a MD simulation and a free-energy perturbation (FEP)-based scanning calculation. Due to *E*-Methyl 2-(2-((benzo- thiazol-2-ylthio)methyl)phenyl)-3-methoxyacrylate (compound **1**) was identified previously as a hit compound targeting cytochrome *bc*_1_ complex (*K*_i_ = 31.10 nM, against porcine *bc*_1_)^21^, the MD simulation was first performed on compound **1**-bound *bc*_1_ complex system. To evaluate the convergence of the MD simulation, plots of root-mean-square deviation (RMSD) and the distances of hydrogen bonds were examined (Fig. S1).

Due to direct interactions of the substituent with the phenyl ring of phenylalanine, electron-donating and electron-withdrawing substituent can effectively increase the π-π stacking interactions^22^. Hence, some typical groups (*a*: −F, *b*: −Cl, *c*: −Br, *d*: −NO_2_, *e*: −CH_3_, *f*: −CF_3_, *g*: −OCH_3_, *h*: −OH, *i*: −NH_2_, *j*: −COOH) were introduced one by one onto different sites of the benzothiazole ring of **1** to produce compounds **2**–**41** and its nitrogen analogues **42**–**81**, respectively (Table 1). To avoid excessive perturbation in FEP-based calculation, only single substitutions were investigated in the present study. In total, 80 computational substitutions were made and the computational changes in binding free energies as a result of each substitution were calculated for each compound. Detailed results of the binding free-energy changes are provided in Table S1.

A 10-fold improvement of potency should be the minimum standard for an acceptable H2L optimization process. Thus, by setting the criterion of the calculated binding free-energy shift (∆∆*G*_cal_) lower than −1.37 kcal/mol (~10-fold improvement in activity), 29 compounds were considered as candidates for further chemical synthesis. Considering the feasibility of organic synthesis, we only synthesized 10 out of them which are easy to synthesize. Their experimental binding free-energy shifts (∆∆G_exp_) ranged from −2.69 to −1.40 kcal/mol. For an ideal method, it can predict not only compounds with higher potency, but also compounds with lower potency. Hence, a further 11 compounds those ∆∆*G*_cal_ larger than −1.37 kcal/mol and easier to synthesise were also selected randomly for chemical synthesis. The synthetic routes for the total 21 compounds are summarised in Scheme S1. Their chemical structures were characterised by ^1^H and ^13^C NMR spectroscopy, HRMS, elemental analysis, and X-ray diffraction analysis (Fig. S2).

### *In Vitro* Activities and Lead Identification

Inhibitory kinetics is of great importance to understand the inhibitory activity of compounds. The inhibition constants (*K*_i_ = 0.39–159.96 nM) of these compounds with the *bc*_1_ complex were determined according to previously established method^23^ and listed in Table 1. These results indicate that the newly synthesized compounds are specific and effective inhibitors of *bc*_1_ complex. Notably, the experimental and calculated binding free-energy shifts (ΔΔ*G*_exp_ and ΔΔ*G*_cal_, respectively) of these compounds showed good linear correlation (*r*^2^ = 0.8), confirming the predictive accuracy of the CSO method.

Based on the above *in vitro* study, compounds with nanomolar and subnanomolar *K*_i_ values were selected from the total 21 newly synthezised compounds for further studies. Pesticide likeness^24^, defined as a complex balance of various physicochemical properties and structure features, can evaluate whether a molecule is similar to the known pesticides. After the filtration of pesticide likeness, seven compounds were kept to adhere to our previously defined rules for pesticide-likeness including molecular weight (MW) ≤ 435 Da, Clog*P* ≤ 6, number of H-bond acceptors (HBA) ≤ 6, number of H-bond donors (HBD) ≤ 2, number of rotatable bonds (ROB) ≤ 9, and number of aromatic bonds (ARB) ≤ 17 (Table 1)^24^. Finally, based on the results of *in vivo* test, three out of seven compounds were found to display good antifungal activity (>70%) against downy mildew and powdery mildew at the concentration of 200 mg/L (Table S2). Among these three compounds, compound **18** completely inhibited the growth of both species of fungus, outperforming the control fungicide, AZ (which inhibited the growth of powdery mildew, but only reduced the growth of downy mildew by 94%). Therefore, compound **18** was recognized as lead compound for subsequent studies.

### *In Vivo* Antifungal Activity

The above results indicated that compound **18** shows excellent potency towards *bc*_1_ complex, pesticide-like properties, and antifungal activity. For a further validation, the controlling efficacy of compound **18** against downy mildew and powdery mildew disease was tested in greenhouse and field models. Because the pathogens exhibit a specific host for cucumber, the inoculations of *Pseudoperonospora cubensis* (*P. cubensis*) and *Sphaerotheca fuliginea* (*S. fuliginea*) were carried out by spraying a conidial suspension on the seed leaves of cucumber. As expected, the greenhouse test indicated that compound **18** showed excellent fungicidal activity against both downy mildew and powdery mildew diseases. The protective and curative EC_90_ values of compound **18** against *P. cubensis* were 4.29 mg/L and 120.29 mg/L, respectively, and against *S. fuliginea* are 1.89 mg/L and 32.89 mg/L, respectively. As a control, the protective and curative EC_90_ values of AZ against *P. cubensis* are 5.36 mg/L and 50.66 mg/L, respectively, and against *S. fuliginea* are 254.30 mg/L and >500 mg/L, respectively.

**Figure 2:**
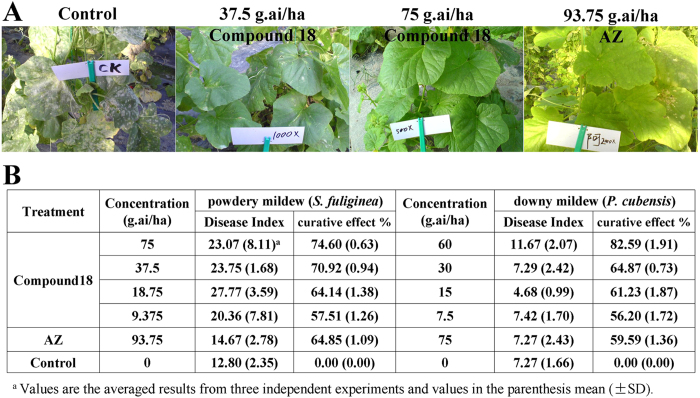
Protection of summer squash and cucumber from attack by *Sphaerotheca fuliginea* and *Pseudoperonospora cubensis*, respectively, by compound 18. (**A**) Compound **18** prevented *S. fuliginea* from infecting and killing summer squash. Compound **18**, at different concentrations, were spread on leaves and photographs were taken after 7 days. (**B**) The relative control effect of compound **18** against powdery mildew and downy mildew in field trials.

To further study the potential of compound **18** against powdery mildew and downy mildew, field experiments were conducted during the growing season of summer squash and cucumber. The summer squash plants at a fruiting stage naturally infected by *S. fuliginea* were used. A foliar application of compound **18** at 75 g.ai/ha recorded 74.60% disease reduction over control. Almost no lesions were observed on the leaves of summer squash. If the treatment concentration of compound **18** was reduced by half to 37.5 g.ai/ha, lesions on the leaves of treated plants expanded very slowly, or ceased to expand, which demonstrates that compound **18** has a curative activity at 37.5 g.ai/ha against the expansion of lesions by *S. fuliginea* with a curative effect of approximately 70.92% (Fig. 2A,B). Lesions on the leaves of control plants, however, expanded quickly, and intense fungal hyphae could be observed. The curative effect is approximately 64.85% for the AZ-treated (93.75 g.ai/ha) summer squash. In addition, field trials for the treatment of downy mildew of cucumber were also performed. Cucumber plants naturally infected by *P. cubensis* were used for the study. Fungi on the leaves of control plants expanded quickly and lesions could be observed. However, we observed that when compound **18** (60 g.ai/ha) was sprayed on the leaves of the cucumber at the primary infection location, only small or no lesions were observed, and the statistical curative effect was approximately 82.59% (Fig. 2B). As a comparison, when leaves of cucumber were treated with AZ (75 g.ai/ha), a lower curative effect (59.59%) was observed. The field experiments show that the curative effect of compound **18** is superior to AZ as a treatment of powdery mildew and downy mildew.

### Action Mechanism

**Figure 3:**
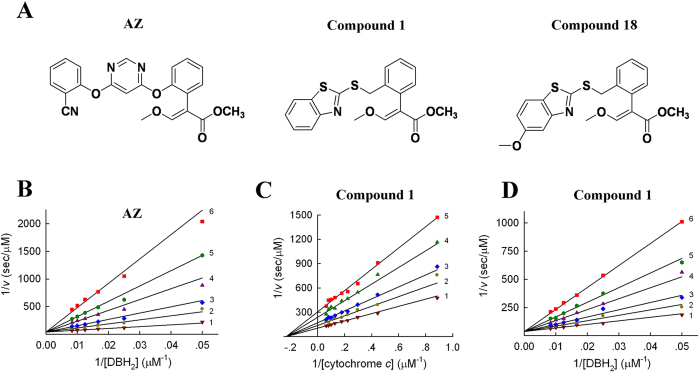
Inhibitory kinetics of *bc*1 complex. (**A**) Chemical structures of AZ (azoxystrobin) and its analogues, compound **1** and compound **18**. These compounds have a common pharmacophore structure: methoxyacrylate (MOA). (**B**) AZ is a competitive inhibitor with respect to substrate DBH_2_. Each reaction mixture contains phosphate buffered saline (PBS; 100 mM, pH 6.5), ethylenediaminetetraacetic acid (EDTA; 2 mM), lauryl maltoside (750 μM), oxidised cytochrome *c* (100 μM), SCR (0.05 nM), DBH_2_ (20–120 μM) and a specified amount of AZ (1, 0 nM; 2, 500 nM; 3, 1000 nM; 4, 2000 nM; 5, 3000 nM; and 6, 5000 nM). (**C**) Compound **1** is non-competitive with respect to cytochrome *c*. The reaction mixture contains 100 mM, pH 7.4), EDTA (0.3 mM), succinate (20 mM), SCR (0.1 nM), cytochrome *c* (1.13–15.6 μM), and a series of concentrations of compound **1** (1, 0 nM; 2, 10 nM; 3, 20 nM; 4, 40 nM; and 5, 60 nM). (**D**) Compound **1** is competitive with respect to DBH_2_. Each reaction mixture contains 100 mM, pH 6.5), EDTA (2 mM), lauryl maltoside (750 μM), oxidised cytochrome *c* (100 μM), SCR (0.05 nM), DBH_2_ (20–120 μM) and a specified amount of compound **1** (1, 0 nM; 2, 50 nM; 3, 100 nM; 4, 200 nM; 5, 300 nM; and 6, 500 nM).

Based on the above *in vitro* and *in vivo* studies, it is reasonable to speculate that compound **18** might have different action mechanism from AZ. Hence, the kinetic effects of compound **18** on cytochrome *bc*_1_ complex were investigated and compared with compound **1** (hit compound) and AZ (Fig. 3A).

Our previous data showed that AZ inhibits the activity of SCR with *K*_i_ = 297.6 ± 7.9 nM, and that AZ is non-competitive with respect to cytochrome *c*^23^. To address the competition of AZ with respect to ubiquinol, we conducted inhibition assays for *bc*_1_ complex (Assay 3) at varying concentrations of DBH_2_ in the absence and presence of the inhibitor AZ. Figure 3B shows a set of double-reciprocal plots of 1/*v*_0_ versus 1/[DBH_2_], which yields a series of straight lines with a common intercept on the 1/*v* axis with different slopes. Because the intercepts of each line on the 1/[DBH_2_] and 1/*v* axes are equal to −1/*K*_m_ and 1/*V*_max_, respectively, at the indicated AZ concentration, we can conclude that *K*_m_ for the DBH_2_ substrate increases with increasing AZ concentration, whereas *V*_max_ is not affected. These results indicate that AZ is a competitive inhibitor of *bc*_1_ complex with respect to substrate DBH_2_, and its kinetic parameters are determined as *K*_m(DBH2)_ = 80.0 ± 7.0 μM and *K*_i_ = 394.7 ± 19.6 nM.

To unravel the inhibitory mechanism of compound **1**, we first examined the dependence of product formation on the concentration of the cytochrome *c* as substrate by monitoring the initial rates of SCR (Assay 1). When the succinate concentration was fixed and that of cytochrome *c* varied in the presence of various fixed concentrations of compound **1**, the double-reciprocal plots yielded a non-competitive pattern (Fig. 3C). Analysis of the data yielded *K*_m_ and *K*_i_ values of 4.3 ± 0.2 μM and 31.1 ± 0.9 nM, respectively. Next, we analysed the effect of ubiquinol as substrate in the DBH_2_-cytochrome *c* system (Assay 3). When DBH_2_ was used as the substrate, a competitive pattern was observed for compound **1**, in which a series of straight lines converged to a point on the vertical axis (Fig. 3D). The *K*_i_ value was determined as 96.5 ± 5.6 nM, which is approximately 3-fold higher than that determined in the succinate-cytochrome *c* system. This difference likely results from the presence of lauryl maltoside in the assay system, as suggested in previous studies^23^. Taken together, compound **1** is a non-competitive inhibitor with respect to substrate cytochrome *c*, but a competitive inhibitor with respect to DBH_2_, similar to fungicide AZ.

**Figure 4:**
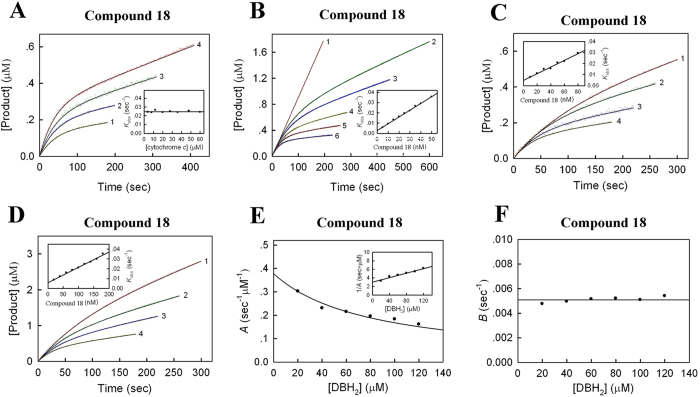
Slow-binding inhibition mechanism. (**A,B**) Effect of various concentrations of cytochrome *c* and compound **18** on the inhibition of SCR activity. Each reaction mixture contains 100 mM, pH 7.4), EDTA (0.3 mM), succinate (20 mM), SCR (0.1 nM), and indicated amounts of cytochrome *c* and compound **18**. The assays were performed in the presence of (**A**) **18** (35 nM) and a specified amount of cytochrome *c* (1, 4 μM; 2, 8 μM; 3, 20 μM; and 4, 48 μM) or (**B**) cytochrome *c* (60 μM) and various concentrations of compound **18** (1, 0 nM; 2, 15 nM; 3, 20 nM; 4, 30 nM; 5, 40 nM; and 6, 50 nM). Experimental data are shown as coloured dots and theoretical values are represented as black solid lines. Insets: plots of *K*_obs_ against concentration of (**A**) cytochrome *c* and (**B**) compound **18**. (**C,D**) Effect of various concentrations of DBH_2_ and **18** on the inhibition of *bc*1 complex activity. Each reaction mixture contains 100 mM, pH 6.5), EDTA (2 mM), lauryl maltoside (750 μM), oxidised cytochrome *c* (100 μM), SCR (0.05 nM), and indicated amounts of DBH_2_ and compound **18**. Two representative sets of inhibitory assays were performed in the presence of (**C**) DBH_2_ (20 μM) and a specified amount of compound **18** (1, 20 nM; 2, 40 nM; 3, 60 nM; 4, 80 nM), or (**D**) DBH_2_ (120 μM) and a specified amount of compound **18** (1, 40 nM; 2, 80 nM; 3, 120 nM; 4, 180 nM). Insets: plots of *K*_obs_ against concentration of compound **18**. (**E**) Plot of the apparent rate constant *A* against concentration of DBH_2_. Inset: Plot of 1/*A* against concentration of DBH_2_. (**F**) Plot of the apparent rate constant *B* against concentration of DBH_2_.

Different with AZ and compound **1**, compound **18** unexpectedly behaves as a slow-binding methoxyacrylate (MOA)-type inhibitor of *bc*_1_ complex (Figs 4A and S3). In the presence of compound **18**, the product formation curve for the SCR-catalysed reaction begins linearly but falls off and approaches a steady state with increasing time. However, when the enzyme was pre-incubated with compound **18** to reach the binding equilibrium, the progress curve simply displays a linear product-versus-time relationship (line 3), with a rate approximately the same as the steady-state rate of line 2 (Fig. S3). These data suggest that compound **18** is a slow-binding inhibitor of *bc*_1_ complex.

To reveal the kinetic mechanism underlying this inhibition, we first examined the effect of the concentration of the cytochrome *c* as substrate and the inhibitor compound **18** on product formation by following the SCR-catalysed reaction (Assay 1). In the presence of different concentrations of cytochrome *c* and a fixed concentration of compound **18**, the time-dependence of the reactions follows the substrate reaction kinetic theory, and the kinetic parameters (the initial [*v*_0_] and steady-state [*v*_s_] rate of the reaction and the observed first-order rate constant [*k*_obs_]) can be determined by nonlinear regression analysis (Fig. 4A). As shown in the inset, the plot of *k*_obs_ against the concentration of compound **18** is a horizontal line, which clearly indicated that compound **18** is non-competitive with respect to cytochrome *c*^23^. Consequently, the values of the true association and dissociation rate constants *k*_+0_ and *k*_−0_ were equal to those of the apparent constants *A* and *B*, respectively, and can be determined from one set of enzymatic assays by varying the concentration of compound **18** at a fixed cytochrome *c* concentration (Fig. 4B). The kinetic parameters of compound **18** were determined, in which the inset shows that *k*_obs_ is proportional to the inhibitor concentration with the slope and the intercept giving *k*_+0_ (=0.00066 ± 0.00001 s^−1^nM^−1^) and *k*_−0_ (=0.00271 ± 0.00031 s^−1^), respectively^23^. The inhibition constant for compound **18** can be calculated as *K*_i_ = *k*_−0_/*k*_+0_ = 4.1 ± 0.5 nM.

Next, we assessed the relationship between compound **18** and the other substrate, ubiquinol, by using the DBH_2_-cytochrome *c* system (Assay 3). At a fixed concentration of compound **18** and different concentrations of DBH_2_, the progress curves exhibited similar curvilinear patterns as observed in the assays at varying cytochrome *c* concentration, as a result of the slow onset of inhibition (Figs S4 and 4A). However, the *k*_obs_ values decreased with increasing concentrations of DBH_2_ (inset of Fig. S4) in contrast to the observation in the inset of Fig. 4A. To achieve a comprehensive understanding, we followed the rate of product formation at various concentrations of DBH_2_ and compound **18**. Two representative sets of curves were shown in Fig. 4C,D, and the *k*_obs_ values at the indicated DBH_2_ concentrations were determined by using the procedures described above. As shown in the insets, both sets of *k*_obs_ values were proportional to the concentration of compound **18**, and the slopes and intercepts of these straight lines denote the apparent association and dissociation constants (*A* and *B*, respectively) at various DBH_2_ concentrations^23^. The characteristic signature of decreasing *A* values and invariable *B* values with increasing concentrations of DBH_2_ suggests that compound **18** is a competitive inhibitor with respect to DBH_2_ (Fig. 4E,F). With the determined *K*_m(DBH2)_ = 80.0 μM, the microscopic inhibition rate constants *k*_+0_ = 0.00038 ± 0.00001 s^−1^nM^−1^ and *k*_−0_ = 0.00509 ± 0.00009 s^−1^, and inhibition constant *K*_i_ = *k*_−0_/*k*_+0_ = 13.4 ± 0.6 nM can be derived. Together, these kinetic analyses suggest that compound **18** is a slow-binding inhibitor of *bc*_1_ complex, and is non-competitive with substrate cytochrome *c* but competitive with respect to DBH_2_.

### Structural Basis of Cytochrome *bc*_1_ Complex

Elucidation of the structural basis of compound **18** inhibition is helpful to further understand its action mechanism and to validate the reliability of the predicted binding modes of inhibitors. Hence, we determined the crystal structures of compound **18** bounds to chicken *bc*_1_ complex at a resolution of 3.23 Å. The binding mode of compound **18** is clearly defined by the electron density map in Fig. S5. According to the crystal structure, compound **18** fits well in the pocket formed by side chains Phe275, Phe129, Ile147, Pro271, Glu272, Leu295, Met125, and Val299. The structural similarity between the predicted and X-ray crystal binding models was found to be RMSD = 0.635 Å (Fig. S6), further validated the prediction capability of CSO method.

**Figure 5:**
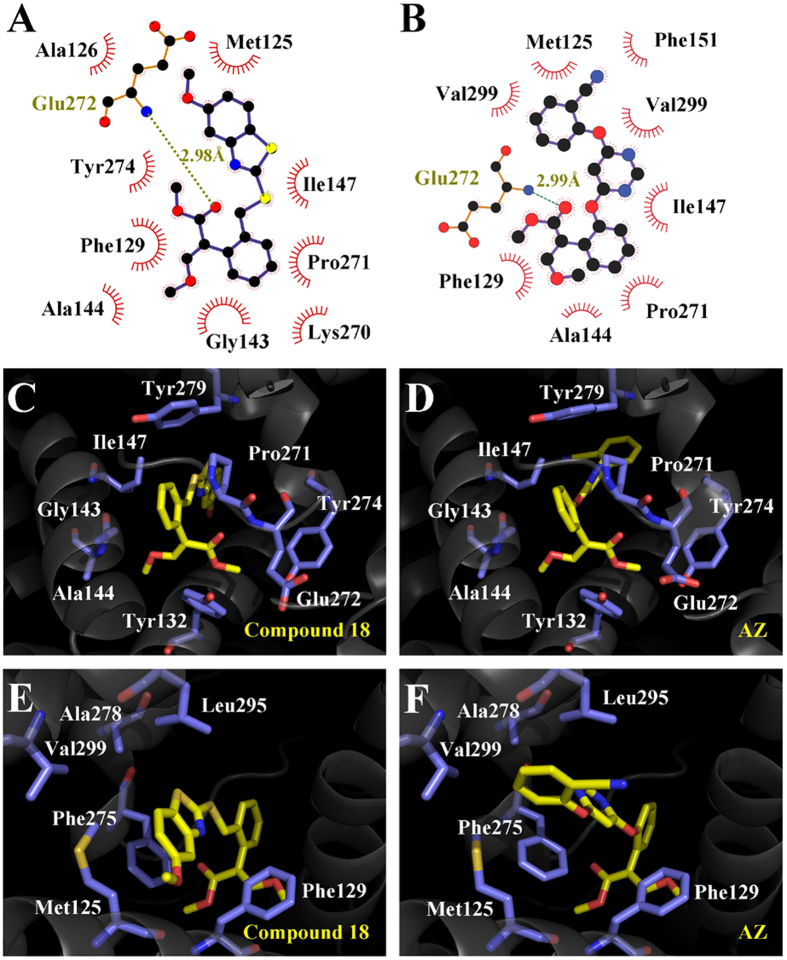
Comparison of binding between compound 18 and AZ. (**A**) Detailed description of the interactions of compound **18** generated with the program Ligplot. Hemispheres represent hydrophobic interactions, whereas lines represent polar interactions. (**B**) Detailed description of the interactions of AZ generated with the program Ligplot. (**C**) The methoxyacrylate and the bridging phenyl ring of compound **18** that binds in a sub-pocket formed by Tyr132, Glu272, Pro271, Tyr279, and Gly143. (**D**) The methoxyacrylate and the bridging phenyl ring of AZ binding in the same sub-pocket with a similar mode. (**E**) The π-π interactions between Phe275 and the benzothiazole side-chain group of compound **18**. (**F**) Distorted π-π interactions between Phe275 and the side-chain group of AZ. Stereo views of the X-ray structure of compound **18** bound to chicken *bc*_1_ complex are available in Fig. S5.

A structural comparison of the binding modes of compound **18** and AZ has revealed interesting similarities and differences. The overall arrangement of the compound **18**-*bc*_1_ complex resembles the structure of the AZ-*bc*_1_ complex with no large conformational changes of the pocket, especially the side chain of the surrounding residues (Fig. 5A,B). Compound **18** mimics the binding mode of AZ, with the methoxyacrylate and the bridging phenyl ring overlapping extensively with that of AZ, both of which bind in a sub-pocket formed by Tyr132, Glu272, Pro271, Tyr279, and Gly143 (Fig. 5C,D). However, notable differences appear in the binding mode of the side chain. The π-π interactions between Phe275 and the benzothiazole side-chain group of compound **18** are improved greatly, relative to that of AZ, because of its large co-planar structure (Fig. 5E,F), which can induce ~73-fold improvement of the *in vitro* activity based on the *K*_i_ value of AZ.

## Discussion

An effective tool to eliminate undesirable compounds prior to expensive and extensive experimental work is crucial for improving the efficiency of H2L optimization. Upon this challenge, CSO method was developed and proved to be an effective method to improve the efficiency of H2L process. Compared with the traditional methods, the CSO method has several advantages. First, CSO method can significantly reduce the numbers of compounds, need to be synthesized. For the present study, we need to synthesize 80 compounds theoretically, but only 21 compounds were synthesized (actually, only ten compounds needed to be synthesized, and the other 11 compounds were just for testing) and 12 compounds with nanomolar to subnanomolar potency were obtained. That means, taking the present study as an example, CSO method improved the efficiency of H2L by about 75%. Second, the CSO method is independent of biological data and molecular descriptors, and has the ability to predict a more expansive chemical space. The high correlation of ΔΔG (*r*^2^ = 0.8 for the linear correlation) and the high correspondence of the predicted and crystallographic poses (RMSD = 0.635 Å) support the reliability and accuracy of the CSO method. On the contrary, the traditional QSAR models are highly dependent on biological data and molecular descriptors, and always used to explain the existing results and have very poor ability to predict the results outside the training sets. Third, because the CSO method is based on the assumption that the binding modes of substituted hits are similar to that of the original hit, it is possible to perform adequate conformational sampling based on the energy-minimized proteins in complex with substituted hits. Therefore, the CSO method is significantly time-saving compared with MD models.

It should be noted that the CSO method also have limitations. Clearly, this method is based on the assumption that the binding modes of substituted hits are similar to that of the original hit. This assumption is reasonable for small substituents. For a more bulky substituent, it may cause a considerable change in the binding mode and hence leads to significantly overestimate of the binding free-energy shift. Therefore, further improvement should be done to make CSO method suitable for broader application (more bulky groups).

The application of CSO method resulted in a candidate (compound **18**). Compared with the commercial fungicide (AZ), the candidate has several advantages as follows: First, compound **18** has a high inhibiting potency for *bc*_1_ complex (*K*_i_ = 4.10 nM), which is up to ~73-fold more potent than AZ (*K*_i_ = 297.6 nM). In the view of ligand efficiency (LE)^25^, compound **18** (LE = 0.45 kcal mol^−1^ per non-H atom) is prior to AZ (LE = 0.3 kcal mol^−1^ per non-H atom). Second, compound **18** exhibited stronger antifungal activity against downy mildew and powdery mildew than AZ. In the greenhouse test and field trial, the protective and curative effect of compound **18** against downy mildew and powdery mildew are much higher than that of AZ. Third, the inhibitory kinetics studies showed that compound **18** exhibit slow-binding characteristics, which is great different from the classical fast-binding of AZ. This significant characteristic may help to overcome the increasing resistance problem that AZ has to face. Finally, the chemical structure of compound **18** is much simple and easily to be synthesized. The successful design of a highly efficient fungicide candidate also demonstrates a promising future of CSO method. In fact, the commercial development of compound **18** in China is in progress.

## Materials and Methods

### Computational protocol

Based on the modification and combination of AutoGrow and the Amber 9.0 program^26^,^27^, the CSO protocol was designed to perform automatically computational substitution, energy minimization, and binding affinity evaluation. Depicted in Fig. 1 is the workflow of the CSO calculations.

### Kinetic assays

The porcine succinate-cytochrome *c* reductase (SCR, the mixture of complex II and *bc*_1_) was prepared essentially according to the previously reported method^28^. The enzymatic activities of SCR, complex II, and *bc*_1_ complex were analyzed in respective reaction mixtures as reported previously^29^. Kinetic analyses for the inhibition mechanism were performed as previously described^23^.

### Greenhouse Fungicidal Activity

The fungicidal activity of compound **18** against *P. cubensis* and *S. fuliginea* was evaluated according to a previously published method^30^ and a potted-plant test method was adopted.

### Field Trials

In the field trials, summer squash plants which is naturally infected by *S. fuliginea* and cucumber plants which is naturally infected by *P. cubensis* were used.

### Crystallization and structure determination

Orthorhombic crystal of chicken *bc*_1_ in the space group P2_1_2_1_2_1_, containing a dimer in the asymmetric unit was prepared under optimized initial crystallization conditions. The structure was validated using the online tools at the molprobity site^31^ and deposited at the protein data bank with ID 4U3F.

## Additional Information

**How to cite this article**: Hao, G.-F. *et al.* Rational Design of Highly Potent and Slow-Binding Cytochrome *bc*_1_ Inhibitor as Fungicide by Computational Substitution Optimization. *Sci. Rep.* **5**, 13471; doi: 10.1038/srep13471 (2015).

## Supplementary Material

Supporting Information

## Figures and Tables

**Table 1:**
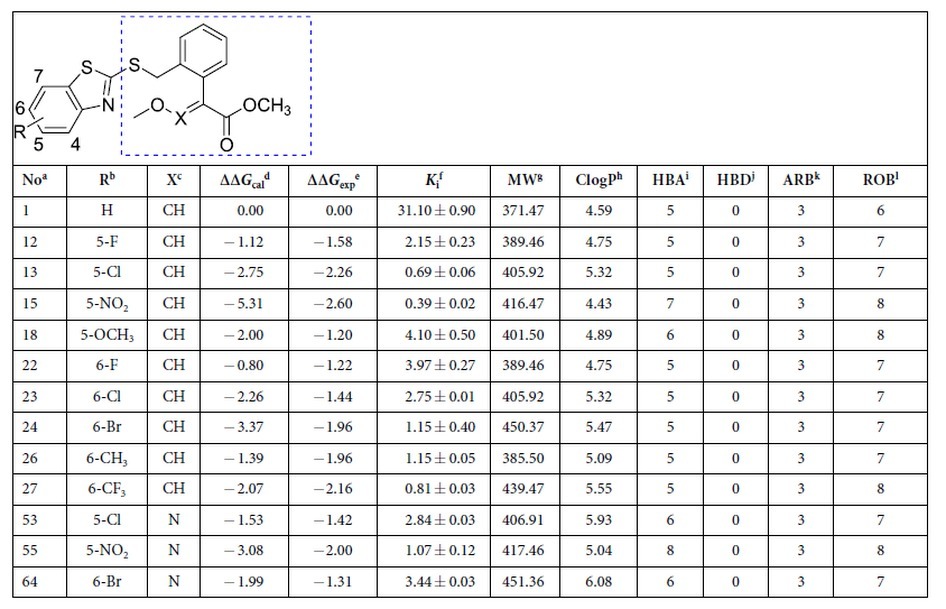
Calculated, experimental binding free energy changes (kcal/mol), inhibition constants (*K*_i_, *n*M), and pesticide-likeness values of compounds with nanomolar inhibition activity.

Precursor hit compound including pharmacophores in the framework and our numbering of the carbon atoms of benzothiazol fragment. The numbers 4–7 denote the sites substituted in this study.

^a^Compounds are named as “series number-site number plus substitution group number” a: −F, b: −Cl, c: −Br, d: −NO_2_, e: −CH_3_, f: −CF_3_, g: −OCH_3_, h: −OH, i: −NH_2_, j: −COOH.

^b^Substitution number and group.

^c^CH is “series 1” and N is “series 2”.

^d^∆∆*G* = ∆*G*(substituent) – ∆*G*(original).

^e^Binding free energy changes are in kcal/mol.

^f^Inhibition constants are in *n*M.

^g^MW, molecular weight.

^h^ClogP: calculated octanol-water partition coefficient.

^i^HBA: number of H-bond acceptors.

^j^HBD: number of H-bond donors.

^k^ROB: number of rotatable bonds.

^l^ARB: number of aromatic bonds.
