# Supplementary material for: Rational Design of Highly Potent and Slow-Binding Cytochrome bc1 Inhibitor as Fungicide by Computational Substitution Optimization
Source: Sci Rep. 2015 Aug 26;5:13471. doi: 10.1038/srep13471 (PMC4549706; doi:10.1038/srep13471)
Supplement: Supporting Information [file srep13471-s1.pdf]

Supporting Information

Rational Design of Highly Potent and Slow-Binding Cytochrome bc_1 Inhibitor as Fungicide by Computational Substitution Optimization

Ge-Fei Hao,[a,†] Sheng-Gang Yang,[a,†] Wei Huang,[a,†] Le Wang,[c] Yan-Qing Shen,[a] Wen-Long Tu,[a] Hui Li,[a] Li-Shar Huang,[d] Jia-Wei Wu,[c] Edward A. Berry,[d] and Guang-Fu Yang[a,b,]*

[a]Key Laboratory of Pesticide & Chemical Biology, Ministry of Education, College of Chemistry, Central China Normal University, Wuhan 430079, P.R.China; [b]Collaborative Innovation Center of Chemical Science and Engineering, Tianjing 300072, P.R.China; [c]MOE Key Laboratory of Protein Sciences, Tsinghua-Peking Center for Life Sciences, School of Life Sciences, Tsinghua University, Beijing 100084, P. R. China; [d]Department of Biochemistry and Molecular Biology, SUNY Upstate Medical University, Syracuse, NY 13210, USA

Correspondence:

Guang-Fu Yang, Ph.D. & Professor

College of Chemistry

Central China Normal University

152 Luoyu Road

Wuhan, Hubei, P. R. China 430079

TEL: 86-27-67867800

FAX: 86-27-67867141

E-mail: gfyang@mail.ccnu.edu.cn

[†]These authors contributed equally to this work.

*To whom correspondence should be addressed. *E-mail:* gfyang@mail.ccnu.edu.cn

Methods

Computational protocol

The computational protocol used in this study is a combination of MD simulation and free energy perturbation (FEP)-based scanning calculation. For predicting the substitution-caused shift of the binding free energy, we first performed MD simulation on the unperturbed system (binding complex of hit molecular with the protein) to obtain the dynamically stable initial structure required for performing the FEP-based CSO calculations. Below, we have described how we carried out the MD and CSO calculations.

To carry out the MD simulations, the topology and coordinate files of the complexes were built with the Leap module of the Amber9 package. Energy minimizations and MD simulations were performed using the Sander module of the Amber9 program. The AMBER ff03 force field was used as the parameters for amino acid residues [1], and the general AMBER force field (gaff) [2] was used for ligand. We tested RESP and AM1-BCC charge methods, but a good linear correlation with experimental data can be obtained by using AM1-BCC (data not shown). Hence, the partial atomic charges of ligand were calculated using the AM1-BCC method implemented in the Antechamber module of the Amber9 package [3]. The counter ions (Na^+) were added to the most electronegative areas around the protein to neutralize the system. All molecules were solvated by a rectangular box of TIP3P waters extended at least 10 Å in each direction from the solute [4]. The cutoff distance for the long-range electrostatic interaction which was treated with particle mesh Ewald (PME) [5,6] and for the van der Waals (vdW) energy terms was set at 10.0 Å. SHAKE algorithm was used to constrain all covalent bonds involving hydrogen atoms [7]. The energy minimization was achieved in three stages. First, movement was allowed only for the water molecules and ions. Next, the backbone atoms of the protein were fixed and the other atoms were allowed to move. Finally, all atoms were permitted to move freely. In each stage, the energy minimization was executed by using the steepest descent method for the first 2000 steps and the conjugated gradient method for the subsequent 3000 steps. Then, the MD simulation was performed according to the following steps. First, the solvent molecules were equilibrated for 10 ps to make sure that the simulated solvent system was in an equilibrated condition. Then the system was gradually heated from 10 to 300 K over 20 ps. Finally to make sure that we obtained a stable MD trajectory for each of the simulated structures, equilibrating calculations were executed for 4 ns at 1 atm and 300 K with applying periodic boundary conditions in the NPT ensemble to avoid edge effects. The time step used for the MD simulations was 2.0 fs. To obtain the best possible binding mode for each ligand, the key intermolecular hydrogen bonds formed after the energy minimization were restrained during the heating and the first 500 ps of the MD simulation at 300 K, and

then the whole complex was relaxed to obtain a stable MD trajectory. During the MD simulation, atomic coordinates were collected every 1 ps.

We saved a total of 100 snapshots from a stable MD trajectory of last 1000 ps, *i.e.* one structure in every 10 ps. In order to calculate the binding free energy change for new ligand with substitution, all force field parameters in the topology files for the hit ligand were replaced with the parameters of the corresponding new ligand by in-house program. To refine the structure, the positions of side chain atoms of all residues were energy-minimized by using the Sander module of Amber9 program *via* a combined use of the steepest descent/conjugate gradient algorithms, with a convergence criterion of 0.1 kcal mol^{-1} $Å^{-1}$ which was appropriate for the desirable computational accuracy. We tried to use different energy minimization methods for the geometry refinement of the snapshot structures and found slight difference for the final energetic results between them (data not shown).

Synthetic chemistry

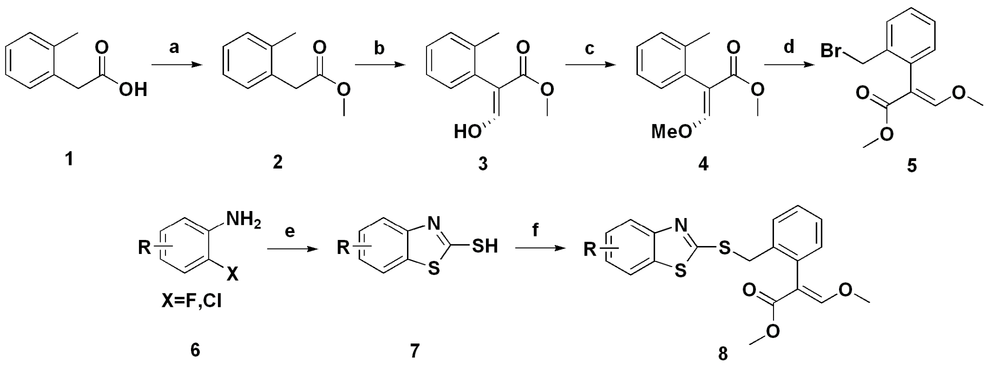

Reagent and Conditions: (a)MeOH, H_2SO_4; (b)HCOOMe, MeONa, PhMe; (c)$(CH_3)_2SO_4$, K_2CO_3; (d)NBS, AIBN, CCl_4, reflux; (e)$C_2H_5OCS_2K$, DMF, reflux; (f)5, K_2CO_3, acetone, reflux.

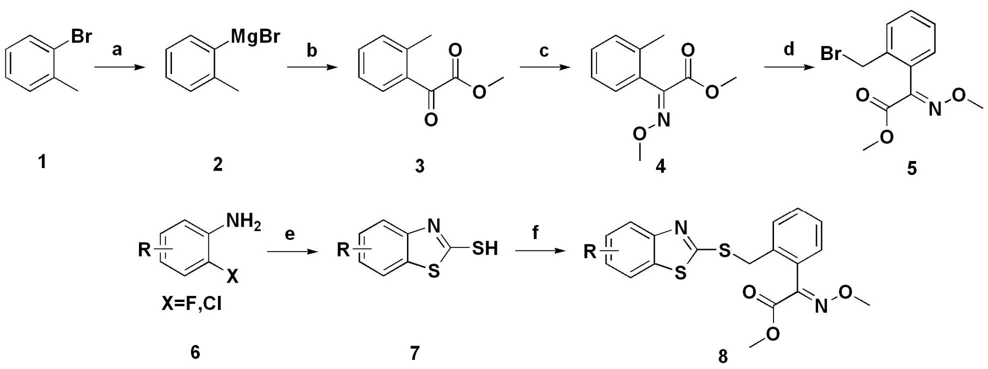

Reagent and Conditions: (a)Mg, THF; (b)$(COOMe)_2$,-78℃; (c)Pyridine, $MeONH_2HCl$; (d)NBS, AIBN, CCl_4, reflux; (e)$C_2H_5OCS_2K$, DMF, reflux; (f)5, K_2CO_3, acetone, reflux.

Scheme S1. General procedure for the synthesis of target compounds.

Unless otherwise noted, all chemical reagents were commercially available and treated with standard methods. Silica gel column chromatography (CC): silica gel (200–300 mesh;

Qingdao Makall Group Co., Ltd; Qingdao; China). Solvents were dried in a routine way and redistilled. ^{1}H NMR and ^{13}C NMR spectra were recorded in $CDCl_3$ or DMSO-d_6 on a Varian Mercury 600 or 400 spectrometer and resonances (δ) are given in ppm relative to tetramethylsilane (TMS). MS spectra were determined using a Trace MS 2000 organic mass spectrometry. Elementary analyses were performed on a Vario EL III elemental analysis instrument. Melting points were taken on a Buchi B-545 melting point apparatus. The product purity was determined by the reverse phase HPLC using an Agilent Eclipse XDB-C_{18} column (5μm, 4.6×150 mm, PN: 993967-902, SN: USKH039322, LN: B07102). The condition were applied, detection = 254 nm; solvent A = CH_3OH; solvent B = H_2O; column temperature: 25 ℃; injection volume: 5 μL; flow: 1mL/min; solvents: A:B = 65:35.

To a mixture of anhydrous K_2CO_3 (0.82 g, 6.0 mmol) in dry acetone (20 mL), 5.0 mmol of the corresponding quinoxaline-2-thiol was added, the resulted mixture was stirred and refluxed for 0.5h, followed by the addition of (*E*)-methyl 2-(2-(bromomethyl)phenyl)-3-methoxyacrylate (1.425 g, 5.0 mmol). The reaction was stirred for an additional 5~8 h under reflux. The resulted mixture was cooled to room temperature and filtered off by suction, and the solvent was evaporated to give the crude product, followed by chromatography on silica using a mixture of petroleum ether and ethyl acetate (10:1) as eluent to give the target compounds.

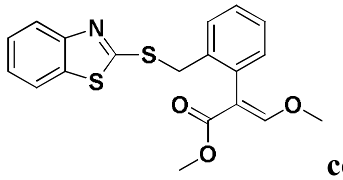

compound 1

Data for compound 1: white soild, yield 70%, HPLC purity > 99%, mp 84-86 ^{o}C; ^{1}H NMR (600 MHz, $CDCl_3$): δ 7.88 (d, J = 8.1 Hz, 1H), 7.74 (d, J = 7.9 Hz, 1H), 7.59 (s, 1H), 7.56 (d, J = 3.9 Hz, 1H), 7.42 (t, J = 7.7 Hz, 1H), 7.33–7.27 (m, 3H), 7.19–7.14 (m, 1H), 4.55 (s, 2H), 3.84 (s, 3H), 3.70 (s, 3H).EI MS: m/z 371(M^+,2), 339(12), 294(28), 236(94), 205(8), 145(100), 131(20), 103(22). ^{13}C NMR (101 MHz, $CDCl_3$): δ 167.79, 167.29, 160.44, 152.93, 135.19, 135.11, 132.71, 131.28, 129.97, 128.25, 127.70, 125.99, 124.14, 121.29, 120.93, 109.89, 61.97, 51.69, 35.73. Anal. Calcd for $C_{19}H_{17}NO_3S_2$: C, 61.43; H, 4.61; N, 3.77; Found: C, 61.56; H, 4.32; N, 3.66.

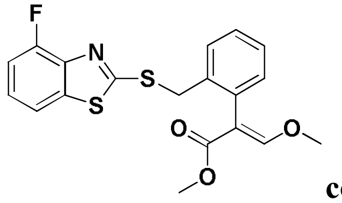

compound 2

Data for compound 2: yellow soild, yield 59%, HPLC purity > 99%, mp 129-131 ^{o}C; ^{1}H NMR (400 MHz, DMSO): δ 7.84 (t, J = 8.9 Hz, 1H, =CH-OCH3), 7.74–7.61 (m, 1H), 7.62–7.48 (m, 1H), 7.46–7.21 (m, 4H,), 7.19–6.99 (m, 1H), 4.54 (s, 2H, CH_2), 4.00–3.73 (m,

3H,), 3.71–3.46 (m, 3H). EI MS: m/z 388(M^+,2), 357(13), 253 (100), 205(10), 145(88), 131(18), 103(19). ^{13}C NMR (101 MHz, $CDCl_3$): δ 168.18, 167.72, 160.43, 155.40, 152.86, 137.69, 134.92, 132.73, 131.26, 129.95, 128.14, 127.71, 124.79, 124.73, 116.54, 111.92, 111.74, 109.72, 61.93, 51.60, 35.83. Anal. Calcd for $C_{19}H_{16}FNO_3S_2$: C, 58.59; H, 4.14; N, 3.60; Found: C, 58.77; H, 3.96; N, 3.66.

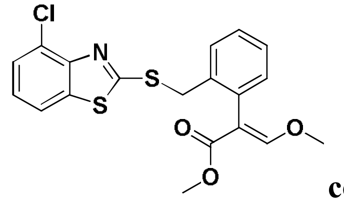

compound 3

Data for compound 3: white soild, yield 68%, HPLC purity > 99%, mp 142-143°C; ^1H NMR (400 MHz, DMSO): δ 7.99 (d, J = 8.1 Hz, 1H, =CH-OCH3), 7.67 (s, 1H), 7.58 (d, J = 7.7 Hz, 2H), 7.36 (t, J = 7.9 Hz, 1H), 7.33–7.25 (m, 2H), 7.11 (s, 1H), 4.52 (s, 2H), 3.85 (s, 3H), 3.59 (s, 3H). EI MS: m/z 405(M^+,7), 373(25), 371(27), 205(41), 145(100), 101(11). ^{13}C NMR (101 MHz,$CDCl_3$): δ 168.62, 167.76, 160.37, 149.91, 136.53, 135.13, 132.67, 131.24, 130.18, 128.17, 127.72, 126.21, 125.99, 124.53, 119.36, 109.89, 62.00, 51.67, 35.83. Anal. Calcd for $C_{19}H_{16}ClNO_3S_2$: C, 56.22; H, 3.97; N, 3.45; Found: C, 55.97; H, 4.13; N, 3.47.

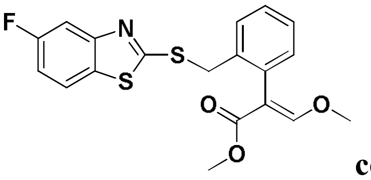

compound 12

Data for compound 12: yellow soild, yield 67%, HPLC purity > 99%, mp 115-117 °C; ^1H NMR (600 MHz, $CDCl_3$): δ 7.65 (dd, J = 8.6, 5.1 Hz, 1H), 7.60 (s, 1H), 7.56 (dd, J = 8.5, 6.2 Hz, 2H), 7.34–7.28 (m, 2H), 7.19–7.13 (m, 1H), 7.09–7.03 (m, 1H), 4.54 (s, 2H), 3.86 (s, 3H), 3.71 (s, 3H). EI MS: m/z 389(M^+,2), 357(12), 312(24), 254(81), 205(7), 73(9), 145 (100), 131(24), 103(22). ^{13}C NMR (101 MHz, $CDCl_3$): δ 171.41, 163.20, 151.77, 149.02, 136.66, 134.20, 130.54, 130.45, 129.70, 128.56, 127.91, 127.05, 126.72, 126.62, 124.69, 123.44, 121.98, 116.93, 63.93, 53.14, 35.63. Anal. Calcd for $C_{19}H_{16}FNO_3S_2$: C, 58.59; H, 4.14; N, 3.60; Found: C, 58.76; H, 3.90; N, 3.66.

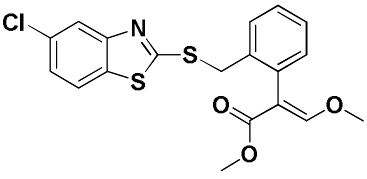

compound 13

Data for compound 13: white soild, yield 77%, HPLC purity > 99%, mp 106-107 °C; ^1H NMR (600 MHz, $CDCl_3$): δ 7.86 (d, J = 1.9 Hz, 1H), 7.64 (d, J = 8.5 Hz, 1H), 7.60 (s, 1H), 7.55 (s, 1H), 7.31 (s, 2H), 7.27 (s, 1H), 7.16 (s, 1H), 4.54 (s, 2H), 3.86 (s, 3H), 3.71 (s, 3H). EI MS: m/z 405(M^+,2), 328(19), 270(67), 145(100), 131(21), 103(21). ^{13}C NMR (101 MHz, $CDCl_3$): δ 169.41, 167.70, 160.37, 153.72, 134.96, 133.35, 132.65, 131.92, 131.26, 129.90, 128.21, 127.72, 124.37, 121.49, 121.11, 109.83, 61.95, 51.66, 35.66. ^{13}C NMR (101 MHz, $CDCl_3$): δ 169.41, 167.70, 160.37, 153.72, 134.96, 133.35, 132.65, 131.92, 131.26, 129.90, 128.21, 127.72, 124.37, 121.49, 121.11, 109.83, 61.95, 51.66, 35.66. Anal. Calcd for $C_{19}H_{16}ClNO_3S_2$: C, 56.22; H, 3.97; N, 3.45; Found: C, 56.48; H, 4.15; N, 3.51.

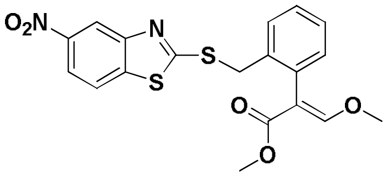

compound 15

Data for compound 15: yellow soild, yield 38%, HPLC purity > 99%, mp 140-142 °C; ^{1}H NMR (600 MHz, $CDCl_3$): δ 8.69 (s,1H), 8.17 (d, J = 8.7 Hz, 1H), 7.84 (d, J = 8.8 Hz, 1H), 7.61 (s, 1H), 7.57 (s, 1H), 7.31 (s, 2H), 7.17 (s, 1H), 4.59 (s, 2H), 3.87 (s, 3H), 3.72 (s, 3H). EI MS: m/z 416(M^+,3), 384(8), 339(31), 28(43), 145(100), 103(22). ^{13}C NMR (101 MHz, $CDCl_3$): δ 171.43, 167.70, 160.44, 152.81, 146.55, 142.01, 134.69, 132.71, 131.34, 129.92, 128.27, 127.88, 121.13, 118.60, 116.29, 109.80, 62.04, 51.72, 35.69. Anal. Calcd for $C_{19}H_{16}N_2O_5S_2$: C, 54.79; H, 3.87; N, 6.73; Found: C, 54.47; H, 3.69; N, 6.62.

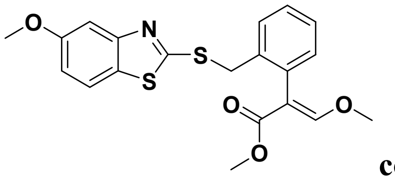

compound 18

Data for compound 18: yellow soild, yield 67%, HPLC purity > 99%, mp 115-117 °C; ^{1}H NMR (600 MHz, $CDCl_3$): δ 7.65 (dd, J = 8.6, 5.1 Hz, 1H), 7.60 (s, 1H), 7.56 (dd, J = 8.5, 6.2 Hz, 2H), 7.34–7.28 (m, 2H), 7.19–7.13 (m, 1H), 7.09–7.03 (m, 1H), 4.54 (s, 2H), 3.86 (s, 3H), 3.71 (s, 3H). EI MS: m/z 389(M^+,2), 357(12), 312(24), 254(81), 205(7), 73(9), 145 (100), 131(24), 103(22). ^{13}C NMR (101 MHz, $CDCl_3$): δ 171.41, 163.20, 151.77, 149.02, 136.66, 134.20, 130.54, 130.45, 129.70, 128.56, 127.91, 127.05, 126.72, 126.62, 124.69, 123.44, 121.98, 116.93, 63.93, 53.14, 35.63. Anal. Calcd for $C_{19}H_{16}FNO_3S_2$: C, 58.59; H, 4.14; N, 3.60; Found: C, 58.76; H, 3.90; N, 3.66.

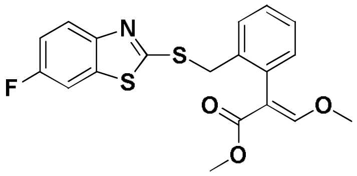

compound 22

Data for compound 22: white soild, yield 59%, HPLC purity > 99%, mp 109-111 °C; ^{1}H NMR (600 MHz, $CDCl_3$): δ 7.86 (d, J = 1.9 Hz, 1H), 7.64 (d, J = 8.5 Hz, 1H), 7.60 (s, 1H), 7.55 (s, 1H), 7.31 (s, 2H), 7.27 (s, 1H), 7.16 (s, 1H), 4.54 (s, 2H), 3.86 (s, 3H), 3.71 (s, 3H). EI MS: m/z 405(M^+,2), 328(19), 270(67), 145(100), 131(21), 103(21). ^{13}C NMR (101 MHz, $CDCl_3$): δ 167.88, 166.87, 160.88, 160.51, 158.44, 149.32, 134.95, 132.65, 131.28, 129.92, 128.23, 127.76, 121.97, 114.44, 109.78, 107.52, 107.25, 62.00, 51.74, 35.86. Anal. Calcd for $C_{19}H_{16}FNO_3S_2$: C, 58.59; H, 4.14; N, 3.60; Found: C, 58.38; H, 3.87; N, 3.81.

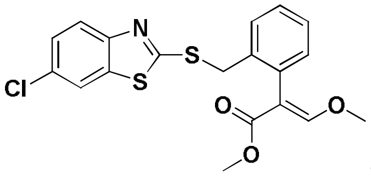

compound 23

Data for compound 23: white soild, yield 71%, HPLC purity > 99%, mp 120-121 °C; ^{1}H

NMR (600 MHz, $CDCl_3$): δ 7.79 (d, J = 8.6 Hz, 1H), 7.71 (s, 1H), 7.61 (s, 1H), 7.55 (s, 1H), 7.38 (d, J = 8.4 Hz, 1H), 7.31 (s, 2H), 7.16 (s, 1H), 4.54 (s, 2H), 3.86 (s, 3H), 3.71 (s, 3H). EI MS: m/z 405(M^+,3), 328(20), 270(48), 205(8), 201(16), 145(100), 131(19), 103(22). ^{13}C NMR (101 MHz, $CDCl_3$): δ 168.33, 167.76, 160.47, 150.95, 135.97, 134.75, 132.70, 131.31, 130.04, 129.93, 128.26, 127.82, 126.78, 121.73, 120.55, 109.78, 62.03, 51.73, 35.96. Anal. Calcd for $C_{19}H_{16}ClNO_3S_2$: C, 56.22; H, 3.97; N, 3.45; Found: C, 55.97; H, 4.13; N, 3.72.

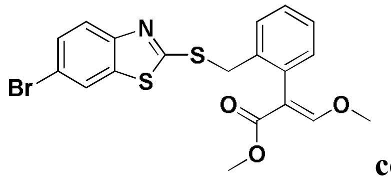

compound 24

Data for compound 24: white soild, yield 65%, HPLC purity > 99%, mp 81-83°C; ^1H NMR (400 MHz, DMSO): δ 8.30 (s, 1H), 7.80 (d, J = 8.6 Hz, 1H), 7.68–7.60 (m, 2H), 7.53 (s, 1H), 7.33–7.25 (m, 2H), 7.10 (d, J = 5.0 Hz, 1H), 4.49 (s, 2H), 3.84 (s, 3H), 3.60 (s, 3H). EI MS: m/z 450(M^+,2), 418(8), 373(22), 314(48), 205(10), 145(100), 131(19), 103(22). ^{13}C NMR (101 MHz, $CDCl_3$): δ 168.27, 167.71, 160.43, 151.50, 136.55, 134.80, 132.69, 131.29, 129.92, 129.43, 128.25, 127.80, 123.41, 122.17, 117.53, 109.80, 61.99, 51.69, 35.84. Anal. Calcd for $C_{19}H_{16}BrNO_3S_2$: C, 50.67; H, 3.58; N, 3.11; Found: C, 50,79; H, 3.49; N, 3.31.

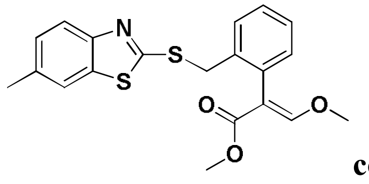

compound 26

Data for compound 26: white soild, yield 35%, HPLC purity > 99%, mp 82-84 °C; ^1H NMR (600 MHz, $CDCl_3$): δ 7.80 (d, J = 8.3 Hz, 1H), 7.59 (s, 1H), 7.57–7.52 (m, 2H), 7.30 (s, 2H), 7.24 (d, J = 8.3 Hz, 1H), 7.15 (s, 1H), 4.55 (s, 2H), 3.85 (s, 3H), 3.70 (s, 3H), 2.46 (s, 3H). EI MS: m/z 385(M^+,3), 308(29), 250(89), 205(11), 145(100), 131(18), 103(21). ^{13}C NMR (101 MHz, $CDCl_3$): δ 167.78, 166.03, 160.41, 150.88, 135.19, 134.22, 132.66, 131.22, 129.94, 128.22, 127.66, 127.43, 120.75, 109.87, 61.97, 51.68, 35.82, 21.37. Anal. Calcd for $C_{20}H_{19}NO_3S_2$: C, 62.31; H, 4.97; N, 3.63; Found: C, 62.53; H, 4.77; N, 3.69.

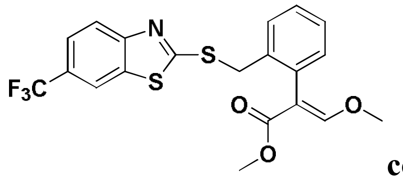

compound 27

Data for compound 27: yellow soild, yield 70%, HPLC purity > 99%, mp 110-111 °C; ^1H NMR (600 MHz, $CDCl_3$): δ 8.02 (s, 1H), 7.96 (d, J = 8.6 Hz, 1H), 7.70–7.65 (m, 1H), 7.62 (s, 1H), 7.57 (s, 1H), 7.31 (s, 2H), 7.18 (s, 1H), 4.58 (s, 2H), 3.87 (s, 3H), 3.72 (s, 3H). EI MS: m/z 439(M^+,2), 407(13), 362(22), 304(65), 205(10), 145(100), 103(22). ^{13}C NMR (101 MHz, $CDCl_3$): δ 171.05, 168.06, 160.65, 154.73, 142.64, 135.15, 134.79, 132.68, 131.36, 129.96, 128.25, 127.86, 126.27, 125.94, 125.40, 123.98, 123.08, 122.70, 121.29, 118.40, 111.98, 109.75, 62.06, 51.87, 35.80. Anal. Calcd for $C_{20}H_{16}F_3NO_3S_2$: C, 54.66; H, 3.67; N, 3.19; Found: C, 54.44; H, 3.40; N, 3.48.

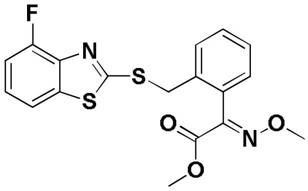

compound 42

Data for compound 42: white soild, yield 67%, HPLC purity > 99%, mp 116-118 °C; ^{1}H NMR (600 MHz, $CDCl_3$): δ 7.61 (d, J = 7.6 Hz, 1H), 7.50 (d, J = 7.9 Hz, 1H), 7.40–7.33 (m, 2H), 7.25–7.20 (m, 1H), 7.18–7.10 (m, 2H), 4.54 (s, 2H), 4.08 (s, 3H), 3.87 (s, 3H). EI MS: m/z 390(M^+,2), 359(21), 344(100), 327(17), 205(82), 175(26), 146(38), 131(65), 116(93). ^{13}C NMR (101 MHz, $CDCl_3$): δ 167.13, 163.18, 155.53, 153.00, 148.95, 141.57, 137.86, 134.40, 130.43, 130.18, 129.65, 128.48, 127.71, 124.96, 124.89, 116.62, 111.97, 111.79, 63.84, 53.06, 35.51. Anal. Calcd for $C_{18}H_{15}FN_2O_3S_2$: C, 55.37; H, 3.87; N, 7.17; Found: C, 55.14; H, 3.62; N, 6.99.

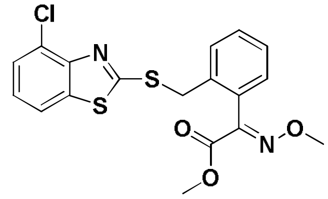

compound 43

Data for compound 43: white soild, yield 41%, HPLC purity > 99%, mp 138-140 °C; ^{1}H NMR (600 MHz, $CDCl_3$): δ 7.69 (s, 1H), 7.62 (d, J = 8.5 Hz, 1H), 7.44 (d, J = 8.0 Hz, 1H), 7.37 (d, J = 21.1 Hz, 3H), 7.19 (d, J = 29.5 Hz, 1H), 4.55 (s, 2H), 4.09 (s, 3H), 3.87 (s, 3H). EI MS: m/z 406(M^+,2), 375(20), 360(100), 205(84), 175(23), 146(43), 131(74), 116(82). ^{13}C NMR (101 MHz, $CDCl_3$): δ 167.66, 163.20, 149.76, 149.07, 136.58, 134.57, 130.49, 130.40, 129.62, 128.44, 127.70, 126.26, 126.15, 124.70, 119.41, 63.89, 53.08, 35.53. Anal. Calcd for $C_{18}H_{15}ClN_2O_3S_2$: C, 53.13; H, 3.72; N, 6.88.; Found: C, 53.45; H, 3.60; N, 6.63.

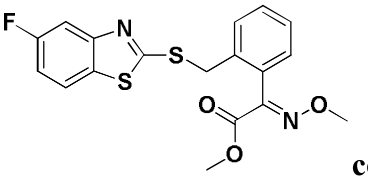

compound 52

Data for compound 52: yellow soild, yield 68%, HPLC purity > 99%, mp 113-114 °C; ^{1}H NMR (600 MHz, $CDCl_3$): δ 7.65 (dd, J = 8.5, 5.1 Hz, 1H), 7.58 (dd, J = 16.0, 8.5 Hz, 2H), 7.37 (dt, J = 14.8, 7.4 Hz, 2H), 7.16 (d, J = 7.4 Hz, 1H), 7.06 (t, J = 8.7 Hz, 1H), 4.49 (s, 2H), 4.08 (s, 3H), 3.87 (s, 3H). EI MS: m/z 390(M^+,3), 344(99), 205(100), 175(23), 146(40), 131(60), 116(77). ^{13}C NMR (101 MHz, $CDCl_3$): δ 168.98, 163.19, 162.89, 160.47, 153.77, 153.65, 148.99, 134.50, 130.47, 130.37, 130.04, 129.72, 128.49, 127.72, 121.52, 121.42, 112.72, 112.48, 108.01, 107.77, 63.87, 53.09, 35.38. Anal. Calcd for $C_{18}H_{15}FN_2O_3S_2$: C, 55.37; H, 3.87; N, 7.17; Found: C, 55.50; H, 3.61; N, 7.36.

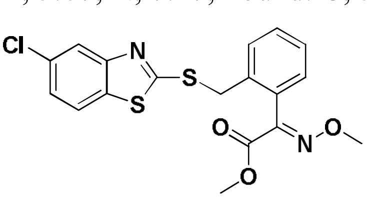

compound 53

Data for compound 53: yellow soild, yield 56%, HPLC purity > 99%, mp 105-107 °C; ^{1}H

NMR (600 MHz, $CDCl_3$): δ 7.61 (d, J = 7.6 Hz, 1H), 7.50 (d, J = 7.9 Hz, 1H), 7.40–7.33 (m, 2H), 7.25–7.20 (m, 1H), 7.18–7.10 (m, 2H), 4.54 (s, 2H), 4.08 (s, 3H), 3.87 (s, 3H).EI MS: m/z 407(M^+,2), 375(19), 205(100), 175(25), 146(32), 131(53), 116(82). ^{13}C NMR (101 MHz, $CDCl_3$): δ 171.40, 163.20, 151.76, 149.02, 136.66, 134.20, 130.54, 130.46, 129.70, 128.56, 127.91, 127.05, 126.62, 123.44, 116.93, 63.94, 53.15, 35.65. Anal. Calcd for $C_{18}H_{15}ClN_2O_3S_2$: C, 53.13; H, 3.72; N, 6.88.; Found: C, 53.37; H, 3.51; N, 6.80.

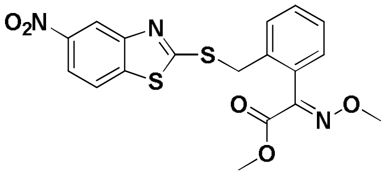

compound 55

Data for compound 55: yellow soild, yield 29%, HPLC purity > 99%, mp 261-263 °C; ^1H NMR (600 MHz, $CDCl_3$): δ 8.69 (s, 1H), 8.18 (d, J = 8.5 Hz, 1H), 7.84 (d, J = 8.6 Hz, 1H), 7.61 (d, J = 7.5 Hz, 1H), 7.42–7.34 (m, 2H), 7.18 (d, J = 7.6 Hz, 1H), 4.54 (s, 2H), 4.10 (s, 3H), 3.90 (s, 3H). EI MS: m/z 417(M^+,3), 386(14), 371(97), 205(100), 175(30), 146(48), 116(10). ^{13}C NMR (101 MHz, $CDCl_3$): δ 170.49, 163.20, 152.72, 148.93, 146.60, 142.05, 134.28, 130.36, 130.04, 129.77, 128.57, 127.85, 121.17, 118.74, 116.46, 63.91, 53.15, 35.34. Anal. Calcd for $C_{18}H_{13}N_3O_5S_2$: C, 51.79; H, 3.62; N, 10.07; Found: C, 52.07; H, 3.87; N, 9.97.

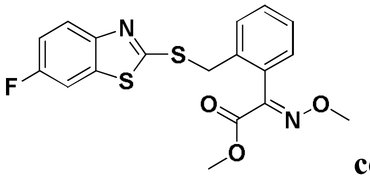

compound 62

Data for compound 62: white soild, yield 64%, HPLC purity > 99%, mp 107-109 °C; ^1H NMR (600 MHz, $CDCl_3$): δ 7.83 (s, 1H), 7.59 (s, 1H), 7.39 (d, J = 16.0 Hz, 3H), 7.17 (s, 2H), 4.49 (s, 2H), 4.08 (s, 3H), 3.87 (s, 3H). EI MS: m/z 390(M^+,5), 359(26), 344(92), 205(100), 185(26), 175(25), 146(36), 131(52), 116(86). ^{13}C NMR (101 MHz, $CDCl_3$): δ 165.85, 163.25, 160.96, 158.53, 149.33, 148.99, 136.14, 136.03, 134.51, 130.36, 130.05, 129.71, 128.50, 127.72, 122.16, 122.07, 114.50, 114.26, 107.56, 107.29, 63.87, 53.11, 35.54. Anal. Calcd for $C_{18}H_{15}FN_2O_3S_2$: C, 55.37; H, 3.87; N, 7.17; Found: C, 55.57; H, 3.61; N, 7.14.

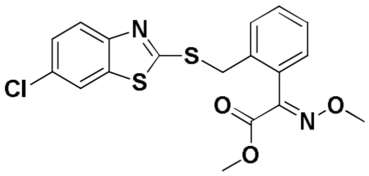

compound 63

Data for compound 63: yellow soild, yield 32%, HPLC purity > 99%, mp 108-110 °C; ^1H NMR (600 MHz, $CDCl_3$): δ 7.78 (d, J = 8.6 Hz, 1H), 7.71 (d, J = 1.8 Hz, 1H), 7.58 (d, J = 7.6 Hz, 1H), 7.37 (p, J = 7.4 Hz, 3H), 7.16 (d, J = 7.3 Hz, 1H), 4.49 (s, 2H), 4.08 (s, 3H), 3.86 (s, 3H). EI MS: m/z 406(M^+,2), 360(59), 205(100), 175(26), 146(38), 131(60), 116(83). ^{13}C NMR (101 MHz, $CDCl_3$): δ 166.95, 163.19, 151.41, 149.00, 136.38, 134.47, 130.39, 130.06, 129.75, 128.51, 127.76, 126.71, 122.04, 120.59, 63.89, 53.11, 35.44. Anal. Calcd for $C_{18}H_{15}ClN_2O_3S_2$: C, 53.13; H, 3.72; N, 6.88.; Found: C, 53.15; H, 3.84; N, 6.87.

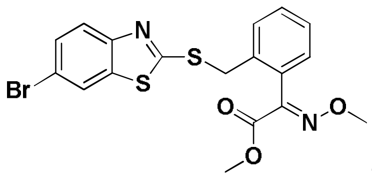

compound 64

Data for compound 64: yellow soild, yield 37%, HPLC purity > 99%, mp 98-100 °C; ^{1}H NMR (600 MHz, $CDCl_3$): δ 7.59 (d, J = 8.3 Hz, 2H), 7.42 – 7.35 (m, 2H), 7.30 – 7.25(m, 1H), 7.25 (d, J = 8.2 Hz, 1H), 7.17 (d, J = 6.4 Hz, 1H), 4.48 (s, 2H), 4.09 (s, 3H), 3.88 (s, 3H). EI MS: m/z 408(M^+,2), 362(93), 205(100), 175(30), 146(51), 131(46), 116(90). ^{13}C NMR (101 MHz, $CDCl_3$): δ 167.03, 163.18, 151.71, 148.97, 136.82, 134.45, 130.37, 130.05, 129.73, 129.38, 128.51, 127.74, 123.44, 122.40, 117.59, 63.87, 53.10, 35.39. Anal. Calcd for $C_{18}H_{15}BrN_2O_3S_2$: C, 47.90; H, 3.35; N, 6.21; Found: C, 48.06; H, 3.26; N, 6.16.

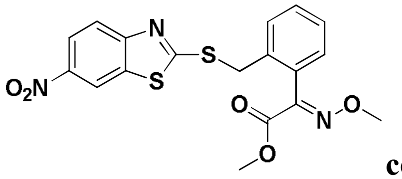

compound 65

Data for compound 65: yellow soild, yield 12%, HPLC purity > 99%, mp 242-244 °C; ^{1}H NMR (600 MHz, $CDCl_3$): δ 8.67 (s, 1H), 8.32 (d, J = 9.6 Hz, 1H), 7.93 (d, J = 9.0 Hz, 1H), 7.61 (d, J = 7.3 Hz, 1H), 7.45–7.35 (m, 2H), 7.18 (d, J = 6.9 Hz, 1H), 4.56 (s, 2H), 4.10 (s, 3H), 3.88 (s, 3H). EI MS: m/z 418(M^+,2), 386(20), 373(14), 371(100), 326(11), 205(99), 175(36), 146(50), 131(56), 116(87). ^{13}C NMR (101 MHz, $CDCl_3$): δ 163.21, 156.70, 148.98, 144.14, 135.69, 134.04, 130.53, 130.11, 129.88, 129.81, 128.66, 128.01, 121.90, 121.28, 117.43, 63.97, 53.19, 35.53. Anal. Calcd for $C_{18}H_{13}N_3O_5S_2$: C, 51.79; H, 3.62; N, 10.07; Found: C, 51.53; H, 3.88; N, 9.98.

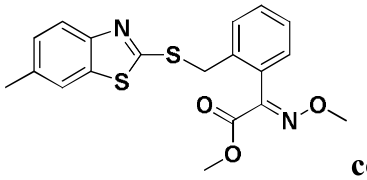

compound 66

Data for compound 66: white soild, yield 30%, HPLC purity > 99%, mp 81-83 °C; ^{1}H NMR (600 MHz, $CDCl_3$): δ 7.81 (d, J = 7.9 Hz, 1H), 7.60 (d, J = 7.0 Hz, 1H), 7.53 (s, 1H), 7.40–7.32 (m, 2H), 7.24 (s, 1H), 7.15 (d, J = 7.3 Hz, 1H), 4.50 (s, 2H), 4.08 (s, 3H), 3.87 (s, 3H), 2.46 (s, 3H). EI MS: m/z 386(M^+,5), 355(32), 340(98), 205(100), 80(26), 175(16), 146(37), 131(51), 116(81). ^{13}C NMR (101 MHz, $CDCl_3$): δ 164.93, 163.19, 150.88, 149.03, 135.33, 134.71, 134.35, 130.34, 130.05, 129.68, 128.41, 127.61, 127.44, 120.90, 120.77, 63.86, 53.06, 35.45, 21.37. Anal. Calcd for $C_{19}H_{18}N_2O_3S_2$: C, 59.05; H, 4.69; N, 7.25; Found: C, 59.29; H, 4.79; N, 7.40.

Kinetic assays

Because it is very difficult to prepare pure bc_1 complex, succinate-cytochrome c reductase (SCR; mixture of respiratory complex II and bc_1 complex) has been widely used for

inhibitory kinetics study of bc_1 inhibitors [8]. Hence, the initial activities of these newly synthesized compounds against bc_1 complex were examined by SCR. As previously described [9], the combined activity of SCR can be determined by using succinate and cytochrome *c* as substrates (Assay 1). We also measured the activity of complex II by using succinate and 2,6-dichloroindophenol (DCIP) as substrates (Assay 2), or the activity of bc_1 complex by using decylubiquinol (DBH_2) and cytochrome *c* as substrates (Assay 3).

The SCR from porcine heart was prepared following the reported protocol [8]. Concentration of cytochrome c_1, estimated from the difference spectra of the ascorbate reduced SCR minus the ferricyanide oxidized form with a extinction coefficient of 17.5 $mM^{-1}cm^{-1}$ for A_{red}^{552} - A_{red}^{540} [10], was used to represent the enzyme concentration. The three redox reactions used in this study were summarized as follows.

Assay 1: $\text{succinate} + \text{cytochrome } c^{3+} \xrightarrow{\text{SCR}} \text{fumarate} + \text{cytochrome } c^{2+}$

Assay 2: $\text{succinate} + \text{DCIP} \xrightarrow{\text{complex II}} \text{fumarate} + \text{DCIPH}_2$

Assay 3: $\text{DBH}_2 + \text{cytochrome } c^{3+} \xrightarrow{bc_1 \text{ complex}} \text{DB} + \text{cytochrome } c^{2+}$

All assays were performed at 23 °C. The enzymatic activity of SCR (Assay 1) was carried out in 1.8 mL of reaction mixture containing 100 mM PBS (pH 7.4), 0.3 mM EDTA, and indicated concentrations of succinate and oxidized cytochrome *c* (Sigma-Aldrich). The activity of succinate-ubiquinone reductase (complex II, Assay 2) was determined in essentially the same reaction mixture, except for the replacement of cytochrome *c* with DCIP (Alfa Aesar). The ubiquinol-cytochrome *c* reductase (bc_1 complex) activity (Assay 3) was examined in 100 mM PBS (pH 6.5), 2 mM EDTA, 750 μM lauryl maltoside (LM, *n*-dodecyl-β-*D*-maltoside, Hampton Research) and indicated concentrations of decylubiquinol (DBH_2) and oxidized cytochrome *c*. DBH_2 was prepared through reduction of decylubiquinone by dithionite (Sigma-Aldrich) as previously described [11,12]. The nonionic detergent LM was used to decrease the interfering non-enzymatic activity though it slightly affects the K_m value of DBH_2 to bc_1 complex [9,13-16]. The reaction was monitored continuously by following the absorbance change at 550 nm or 600 nm on a Perkin-Elmer Lambda 45 spectrophotometer equipped with a magnetic stirrer in the cuvette holder. The extinction coefficients used were 18.5 $mM^{-1}cm^{-1}$ for A_{red-ox}^{550} for cytochrome *c* reduction (Assays 1 and 3) and 21 $mM^{-1}cm^{-1}$ for A_{red-ox}^{600} for DCIP reduction (Assay 2). For each reaction, the non-enzymatic rate was followed for at least 100 sec before the enzymatic reaction was initiated. Kinetic analyses for the inhibition mechanism were performed as previously

described [9]. All of the compounds significantly inhibited the activity of bc_1 complex, as well as the SCR activity, which involves both complex II and bc_1 complex. By contrast, none of the compounds affected complex II activity, even at a high concentration (20 μM).

Greenhouse Fungicidal Activity

The fungicidal activity of compound **18** against P. cubensis and S. fuliginea was evaluated according to a previously published method [17] and a potted-plant test method was adopted. Commercial fungicide AZ was evaluated as a control under the same conditions. Cucumber seeds were soaked in water for 2 h at 50℃ and then were kept moist for 24 h at 28℃ in an incubator. When the radicles grew to 0.5 cm, the seeds were grown in plastic pots containing a 1:1 (v/v) mixture of vermiculite and peat. At the stage of two leaves, all of the cucumber plants used for inoculations were divided into two groups to test for protective and curative effects. In the protective action, compound **18** and commercial fungicide AZ were sprayed on the leaves before the inoculation. However, the fungal inoculation and chemical spraying are reverse in the protective action.

In the protective action, compound 18 and commercial fungicide AZ were sprayed with a hand spray on the surface of the seed leaves at concentrations of 200, 100, 50, 25, 12.5, 6.25, 3.125, 1.5625 mg/L. After 2 h, inoculations of P. cubensis and S. fuliginea were carried out by spraying a conidial suspension (concentration 10^5 conidia ml^{-1}), which was repeated 4 times. After inoculation, the cucumber seedlings were cultured in a culture room at 25±2℃, 70%~80% relative humidity, and 16L:8D photoperiod for 8 d. In the curative action, the fungal inoculation and chemical spraying were reversed with the protective action. Foliar spray with fungicides to inoculated cucumber plants (before disease expression) till near-run-off was made on the 7th and 15th day after emergence. The fungicidal activity was evaluated when the nontreated cucumber plant fully developed symptoms. The area of inoculated and treated leaves covered by disease symptoms was assessed and compared to that of nontreated ones to determine the average disease index.

The disease range (i) was visually evaluated on the first true leaf and ranked as a percentage infected area using a rating of 0, 1, 3, 5, 7, or 9, denoting proportions of disease over the whole leaf area of 0%, < 5%, 6–10%, 11–25%, 26–50%, and > 50%, respectively [18].

The disease index and control effect of compound 18 were calculated via the following equations [19]:

$$\text{Disease index (\%)} = \sum(N_i \times i)/(N \times 9) \times 100$$

$$\text{Protective/curative effect (\%)} = [(CK - PT) / CK] \times 100$$

where Ni is the number of disease plant leaves, is the disease range, N is the total number of plant leaves, CK is the disease index of control and PT is the disease index of treatment.

Field Trials

Field trials were conducted in Ningbo, Zhejiang Province, China, to determine the potential control of S. fuliginea *in* summer squash and P. cubensis *in* cucumber. In the field trials, summer squash plants which is naturally infected by S. fuliginea and cucumber plants which is naturally infected by P. cubensis were used. Each field was divided into 12 plots, and the size of each plot was 4 × 5 m (width × length). The concentrations used were 0, 9.375, 18.75, 37.5, 75 g.ai/ha for compound 18 treatment and 93.75 g.ai/ha for AZ treatment in the curative field trial for S. fuliginea *of* summer squash. The concentrations used were 0, 7.5, 15, 30, 60 g.ai/ha for compound 18 treatment and 75 g.ai/ha for AZ treatment in the curative field trial for P. cubensis *of* cucumber. All field trials were arranged in a block design and each treatment on a given test was replicated a minimum of three times throughout the field. Foliar application of fungicides was made by using a hand sprayers after the emergence of disease. Each treatment took up in four plots and was distributed randomly in each field. The relative control effects were assessed after the 7th day of foliar application [18].

$$\text{Relative control effect (\%)} = [1 - (CK_0 \times PT_1)/(CK_1 \times PT_0)] \times 100$$

where CK_0 is the disease index of control before treatment, CK_1 is the disease index of control after treatment, PT_0 is the disease index of before treatment, and PT_1 is the disease index of after treatment.

Crystallisation and Structure Determination

Orthorhombic crystals of chicken *bc*$_1$ in space group $P2_12_12_1$, that contained a dimer in the asymmetric unit, were prepared by sitting-drop vapour diffusion at 273 K under optimised initial crystallisation conditions with cacodylate (50 mM); TrisHCl (9.4 mM); K-MES (30

mM, pH 6.8); K-MOPS (1.8 mM, pH 7.2); NaCl (30 mM); KCl (31 mM); $MgCl_2$ (10 mM); glycerol (91 g/l); PEG (30 g/l, 4 kDa); NaN_3 (0.9 mM); EDTA (0.05 mM); undecyl maltoside (0.47 g/l); and octyl glucoside (31 mM, pH 6.77). Crystals were grown from chicken bc_1 treated with a two-fold excess of compound **18**. Diffraction data were collected at beam line A1 of the Cornell High Energy Synchrotron Source (CHESS) at an x-ray wavelength of 0.9770 Å and reduced with the HKL package [20]. Data from one crystal extending to 3.23 Å were used to refine the previously determined structure of the protein (3TGU) with the Qo-site ligand removed, using phenix.refine [21]. Electron density maps (2mFo-DFc and mFo-DFc) were calculated and used to guide the placement of the new inhibitor. Refinement was continued with phenix.refine using a geometrical restraints file generated for the inhibitor by phenix.elbow [22]. The structure was validated by using the online tools at the molprobity site [23] and deposited at the protein data bank with ID 4U3F. The statistics of data and structures were summarised in **Table S3**.

As shown in **Fig. 5A**, the pharmacophore of compound **18** was inserted into a slot bound by Phe129, Tyr132, Phe275, and Glu272. Only one direct polar contact was found and it involves the carbonyl oxygen of the methoxyacrylate and the backbone amide of Glu272. The bridging phenyl ring was nearly at right angles to the plane of the methoxyacrylate and was inserted between residues Pro271 and Gly143. The benzothiazole side-chain group that extends from the bridging ring forms π-π stacking interactions with the phenyl ring of Phe275, which is important for tight binding in addition to loose contact with Met125. The methoxyl substituent rests against the C helix, which leads to van der Waals contacts with Met125 and Ala126. On the other side, the methoxy group is overlaid by density assigned to the hydrocarbon tail of a lipid. Adding to this, the other residues in the binding pocket mainly establish extensive hydrophobic interactions with compound **18**.

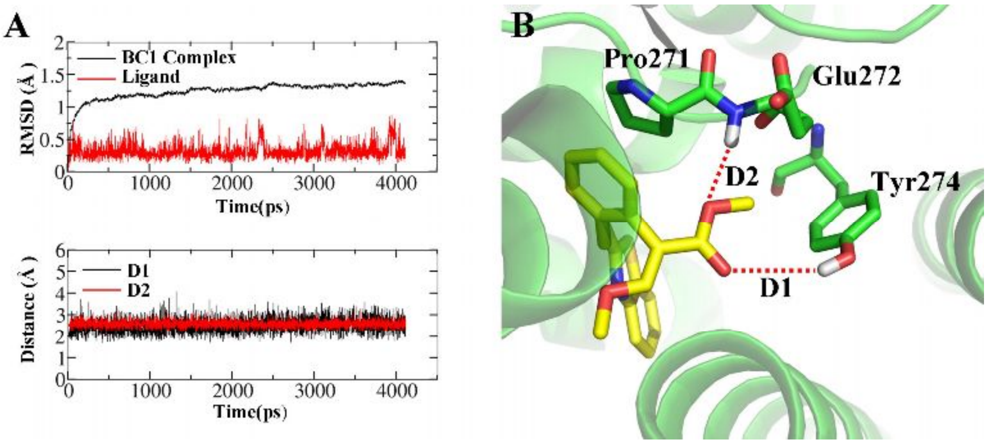

Figure S1 MD trajectories for bc_1 complex binding with hit compound: RMSD representing the root-mean-square deviation of the simulated positions of the protein backbone atoms and inhibitor atoms from those in the initial structure; plots of the key inter-nuclear distances *vs* the simulation time for the inhibitors binding with bc_1 complex.

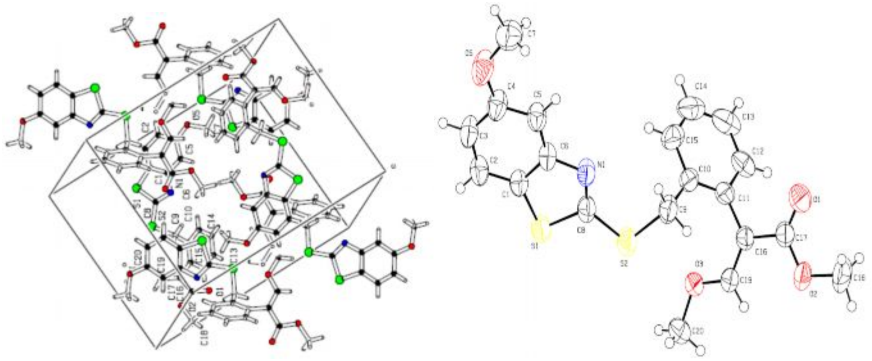

Figure S2 Crystal structure of compound **18**. A colorless block of compound **18** (0.3 mm × 0.2 mm × 0.2 mm) was mounted on a quartz fiber. Cell dimensions and intensities were measured at 298 K on a Bruker SMART CCD area detector diffractometer with graphite-monochromated Mo K radiation (λ = 0.71073 Å); 14987 measured reflections; 4666 independent reflections (R_{int} = 0.0254). Data were corrected for Lorentz and polarization effects and for absorption (T_{min} = 0.9165; T_{max} = 0.9432). The structure was solved by direct methods using SHELXS-97; all other calculations were performed with Bruker SAINT System and Bruker SMART programs. Full-matrix least-squares refinement based on F^2 using the weight of $1/[\sigma^2 (F_o^2) + (0.0934P)^2 + 0.1767P]$ gave final values of R = 0.0767, ωR = 0.1614, and GOF(F) = 1.072. Max/min residual electron density = 0.344/-0.200 e $Å^{-3}$. Hydrogen atoms were observed and refined with a fixed value of their isotropic displacement parameter.

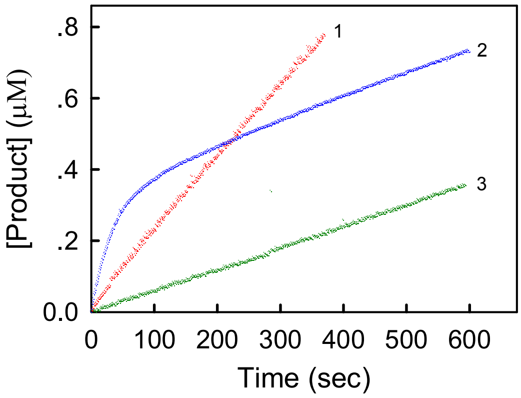

Figure S3 Compound **18** exhibits slow-binding behavior to bc_1 complex. Each reaction mixture contains 100 mM PBS (pH 7.4), 0.3 mM EDTA, 20 mM succinate, 60 μM cytochrome *c*, 0.1 nM porcine SCR, and certain amount of inhibitors (1, 200 nM compound **1**; 2 and 3, 40 nM compound **18**). 1 and 2: reaction initiated with enzyme SCR; 3: enzyme preincubated with compound **18** in the cuvette, and then reaction initiated by adding substrate succinate.

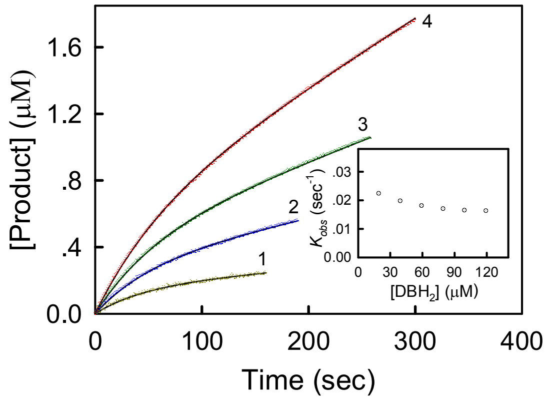

Figure S4 The k_{obs} value decreases with increasing concentrations of DBH_2 at fixed compound 18 concentration. Each reaction mixture contains 100 mM PBS (pH 6.5), 2 mM EDTA, 750 μM lauryl maltoside, 100 μM oxidized cytochrome *c*, 0.05 nM SCR, 60 nM compound 18 and certain amount of DBH_2 (1, 20 μM; 2, 40 μM; 3, 60 μM; 4, 100 μM). Experimental data were shown as colored dots and the theoretical values were indicated by black solid lines. Inset: plot of k_{obs} against concentration of DBH_2.

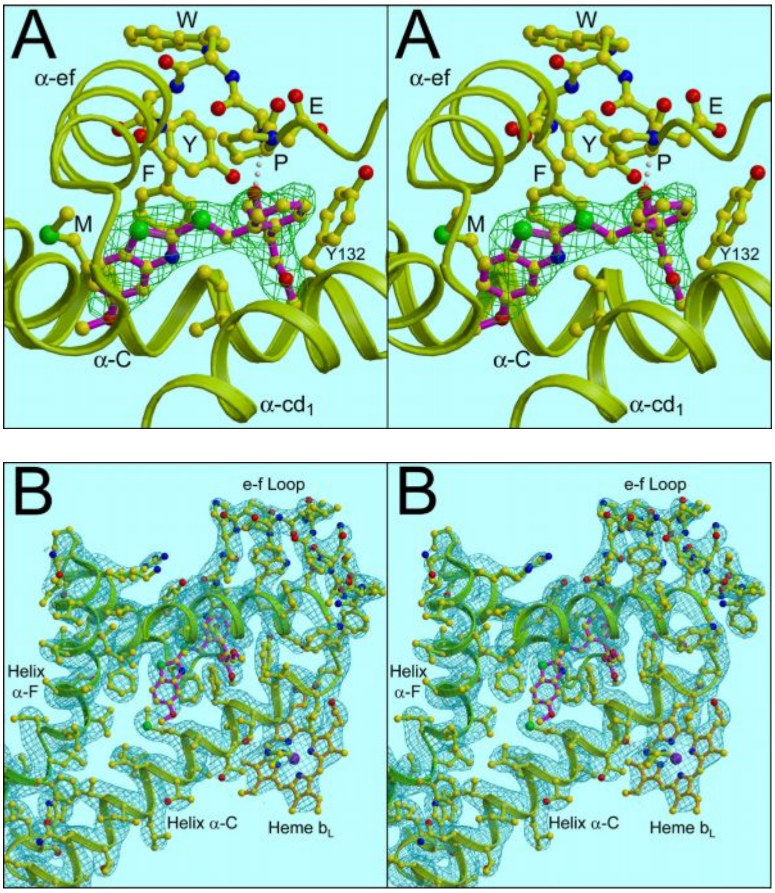

Figure S5 Electron density from X-ray data. **A.** Stereo representation of compound 18 binding site showing omit electron density around the ligand region. The inhibitor was removed from both monomers of the structure and three additional macro-cycles of positional and individual B-factor refinement were performed to eliminate bias from the model. An (mF_o–DF_c) difference map was calculated from the refined structure, and contoured at 0.2 $e^-/Å^3$ (3.2). The positive difference density in the Qo site (green mesh) unambiguously defines the orientation of the inhibitor (ball-and-stick with magenta bonds, superimposed from the final structure with inhibitor). The amino acids of the PEWYF sequence are depicted, as are Met125 (M), Y132, and I147. **B.** A $2mF_o$-DF_c map calculated from the final deposited structure shows the quality of the electron density. Density around helices C, cd_1, ef, and F; and the ef loop, is contoured at 0.3 e^-/Å3 (1.84). Compound 18 is drawn with magenta bonds, and heme b_L with orange bonds. The unlabeled horizontal helix in the upper front center is helix -cd_1. The upper surface surrounded by cd1-cd_1, ef-loop, and the angle between -ef and -F constitutes the IDP-docking crater. Figure made using "O" [24], Molscript [25], and Raster3D [26].

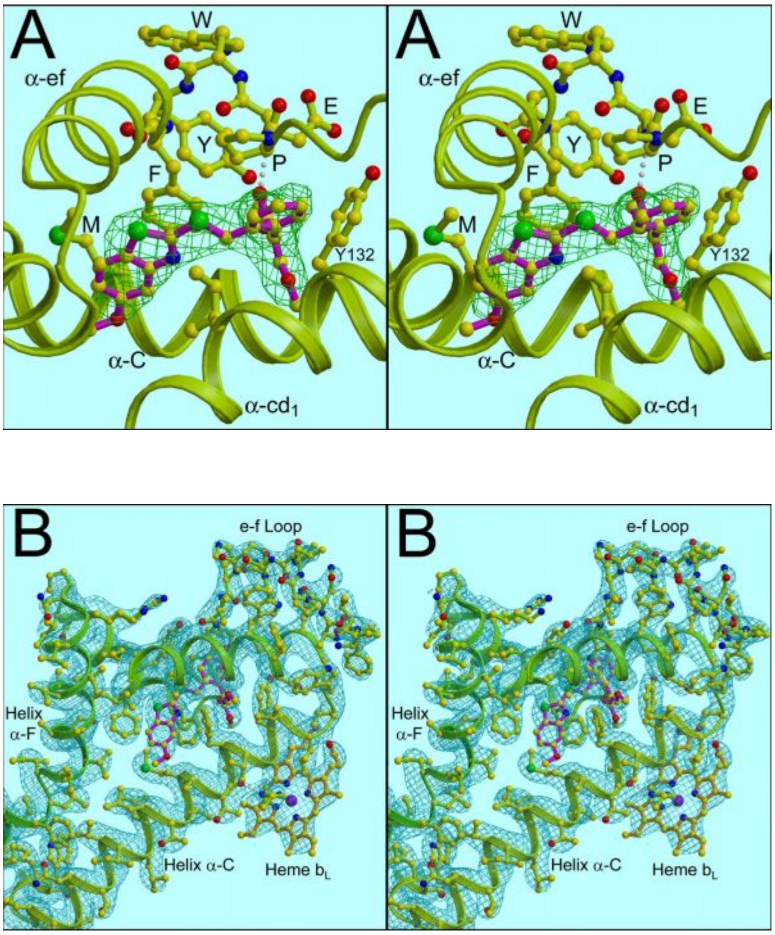

Figure S5x Same as Figure S5 but with stereo pairs reversed for cross-eyed viewing.

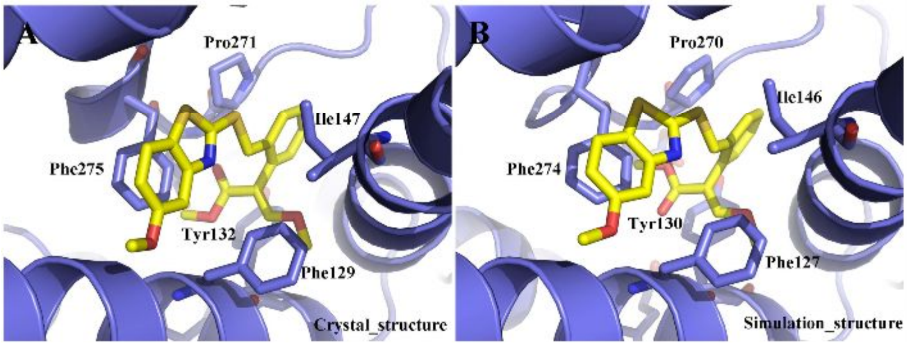

Figure S6 The comparison between the modeling and the X-ray crystal structures of bc_1 in complex with compound 18 (RMSD = 0.635 Å).

Table S1 Calculated, experimental binding free energy changes (kcal/mol), and inhibition constants (K_i, nM) of compounds with bc_1 complex. Precursor compounds including pharmacophores in the framework and our numbering of the carbon atoms of benzothiazol fragment. The numbers 4-7 denote the sites substituted in this study. 29 lead candidates highlighted in blue were further evaluated for chemical synthesis with $\Delta\Delta G_{cal}$ lower than -1.37 kcal/mol. Finally, 10 of them were successfully synthesized. In addition, another 11 compounds with lower predicted activities (highlighted in red) were also randomly synthesized.

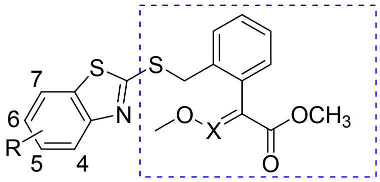

Comp. No[a]	R[b]	X[c]	$\Delta\Delta E_{ele}$[d]	$\Delta\Delta E_{vdw}$	$\Delta\Delta E_{MM}$	$\Delta\Delta G_{pb}$	$\Delta\Delta G_{cal}$[e]	$\Delta\Delta G_{exp}$	K_i[f]
Comp. 1	H	CH	0.00	0.00	0.00	0.00	0.00	0.00	31.10±0.90
Comp. 2	4-F	CH	-0.03	-0.45	-0.47	0.43	-0.04	0.09	35.93±1.24
Comp. 3	4-Cl	CH	-0.07	-1.33	-1.39	1.23	-0.16	0.47	68.55±1.21
Comp. 4	4-Br	CH	-0.21	-1.92	-2.12	1.92	-0.19		
Comp. 5	4-NO_2	CH	-0.54	-4.83	-5.36	3.04	-2.32		
Comp. 6	4-CH_3	CH	0.15	-1.42	-1.26	1.19	-0.06		
Comp. 7	4-CF_3	CH	-0.38	-2.67	-3.04	2.50	-0.54		
Comp. 8	4-OCH_3	CH	0.43	-3.70	-3.26	3.40	0.15		
Comp. 9	4-OH	CH	-0.44	-1.09	-1.52	3.74	2.21		
Comp. 10	4-NH_2	CH	-1.46	-0.84	-2.29	1.95	-0.34		
Comp. 11	4-COOH	CH	-0.83	-3.10	-3.91	3.51	-0.40		
Comp. 12	5-F	CH	-1.47	-0.66	-2.12	1.00	-1.12	-1.58	2.15±0.23
Comp. 13	5-Cl	CH	-1.34	-2.51	-3.84	1.09	-2.75	-2.26	0.69±0.06
Comp. 14	5-Br	CH	-1.57	-3.42	-4.98	1.47	-3.51		
Comp. 15	5-NO_2	CH	-2.72	-5.52	-8.23	2.92	-5.31	-2.60	0.39±0.02
Comp. 16	5-CH_3	CH	-0.07	-2.49	-2.55	0.48	-2.07		
Comp. 17	5-CF_3	CH	-2.55	-3.06	-5.59	2.58	-3.01		
Comp. 18	5-OCH_3	CH	-0.58	-3.32	-3.88	1.88	-2.00	-1.20	4.10±0.50

Comp. 19	5-OH	CH	-0.95	-1.37	-2.31	3.27	0.96		
Comp. 20	5-NH_2	CH	-0.21	-1.41	-1.60	2.11	0.51		
Comp. 21	5-COOH	CH	-4.09	-4.97	-9.05	8.47	-0.58		
Comp. 22	6-F	CH	-0.83	-0.31	-1.14	0.34	-0.80	-1.22	3.97±0.27
Comp. 23	6-Cl	CH	-0.91	-2.08	-2.98	0.72	-2.26	-1.44	2.75±0.01
Comp. 24	6-Br	CH	-1.33	-2.93	-4.26	0.89	-3.37	-1.96	1.15±0.40
Comp. 25	6-NO_2	CH	-2.13	-5.18	-7.30	3.48	-3.82		
Comp. 26	6-CH_3	CH	-0.13	-2.14	-2.25	0.87	-1.39	-1.96	1.15±0.05
Comp. 27	6-CF_3	CH	-1.94	-3.38	-5.31	3.24	-2.07	-2.16	0.81±0.03
Comp. 28	6-OCH_3	CH	-1.33	-2.65	-3.97	2.26	-1.71		
Comp. 29	6-OH	CH	-1.33	-1.18	-2.50	5.20	2.70		
Comp. 30	6-NH_2	CH	-0.53	-1.28	-1.80	2.95	1.15		
Comp. 31	6-COOH	CH	-2.80	-4.44	-7.24	10.67	3.43		
Comp. 32	7-F	CH	0.17	-0.63	-0.44	-0.62	-1.06		
Comp. 33	7-Cl	CH	0.25	-2.77	-2.51	-0.52	-3.03		
Comp. 34	7-Br	CH	0.13	-3.71	-3.58	-0.42	-4.00		
Comp. 35	7-NO_2	CH	0.57	-6.49	-5.92	1.74	-4.17		
Comp. 36	7-CH_3	CH	-0.17	-2.73	-2.88	0.45	-2.43		
Comp. 37	7-CF_3	CH	0.48	-3.35	-2.86	0.76	-2.10		
Comp. 38	7-OCH_3	CH	-0.49	-3.95	-4.43	2.47	-1.97		
Comp. 39	7-OH	CH	-0.75	-1.34	-2.08	6.23	4.15		
Comp. 40	7-NH_2	CH	-0.90	-1.37	-2.26	4.05	1.80		
Comp. 41	7-COOH	CH	-0.63	-3.93	-4.55	7.85	3.31		
Comp. 42	4-F	N	0.62	-0.83	-0.20	1.74	1.54	0.97	159.96±1.10
Comp. 43	4-Cl	N	0.66	-1.73	-1.06	2.19	1.13	0.77	114.29±2.25
Comp. 44	4-Br	N	0.36	-2.33	-1.97	2.61	0.64		
Comp. 45	4-NO_2	N	-0.15	-5.28	-5.42	4.82	-0.61		
Comp. 46	4-CH_3	N	0.65	-1.82	-1.15	2.01	0.86		
Comp. 47	4-CF_3	N	0.45	-3.11	-2.64	3.79	1.15		
Comp. 48	4-OCH_3	N	0.96	-4.07	-3.10	4.32	1.22		
Comp. 49	4-OH	N	-0.03	-1.47	-1.49	4.74	3.26		
Comp. 50	4-NH_2	N	-0.75	-1.23	-1.97	2.86	0.88		
Comp. 51	4-COOH	N	0.04	-3.55	-3.49	4.43	0.94		
Comp. 52	5-F	N	-1.15	-1.02	-2.16	2.00	-0.16	-0.28	19.30±1.32
Comp. 53	5-Cl	N	-0.89	-2.87	-3.75	2.23	-1.53	-1.42	2.84±0.03
Comp. 54	5-Br	N	-1.27	-3.78	-5.05	2.48	-2.57		
Comp. 55	5-NO_2	N	-2.49	-5.93	-8.41	5.33	-3.08	-2.00	1.07±0.12
Comp. 56	5-CH_3	N	0.14	-2.85	-2.70	1.32	-1.37		
Comp. 57	5-CF_3	N	-2.30	-3.43	-5.72	3.70	-2.02		
Comp. 58	5-OCH_3	N	-0.10	-3.66	-3.75	2.96	-0.80		
Comp. 59	5-OH	N	-0.34	-1.72	-2.05	4.37	2.33		
Comp. 60	5-NH_2	N	0.26	-1.74	-1.47	3.34	1.87		

Comp. 61	5-COOH	N	-3.76	-5.33	-9.08	9.63	0.56		
Comp. 62	6-F	N	-0.40	-0.70	-1.08	1.46	0.37	0.55	78.34±1.37
Comp. 63	6-Cl	N	-0.37	-2.46	-2.82	1.85	-0.97	-0.71	9.37±1.24
Comp. 64	6-Br	N	-0.81	-3.30	-4.10	2.11	-1.99	-1.31	3.44±0.03
Comp. 65	6-NO_2	N	-1.80	-5.58	-7.37	6.15	-1.22	-0.08	27.30±1.27
Comp. 66	6-CH_3	N	0.07	-2.52	-2.43	1.88	-0.56	-0.57	11.89±1.08
Comp. 67	6-CF_3	N	-1.69	-3.77	-5.44	4.37	-1.07		
Comp. 68	6-OCH_3	N	-0.97	-2.99	-3.94	3.26	-0.69		
Comp. 69	6-OH	N	-1.01	-1.55	-2.55	6.08	3.53		
Comp. 70	6-NH_2	N	-0.24	-1.64	-1.87	3.75	1.88		
Comp. 71	6-COOH	N	-2.37	-4.82	-7.18	11.84	4.66		
Comp. 72	7-F	N	0.40	-1.04	-0.63	0.49	-0.14		
Comp. 73	7-Cl	N	0.74	-3.18	-2.43	0.66	-1.77		
Comp. 74	7-Br	N	0.46	-4.15	-3.68	0.58	-3.10		
Comp. 75	7-NO_2	N	0.88	-6.92	-6.03	4.13	-1.90		
Comp. 76	7-CH_3	N	0.09	-3.15	-3.05	1.47	-1.58		
Comp. 77	7-CF_3	N	0.86	-3.78	-2.91	1.83	-1.07		
Comp. 78	7-OCH_3	N	-0.27	-4.36	-4.62	3.46	-1.16		
Comp. 79	7-OH	N	-0.18	-1.76	-1.94	7.46	5.53		
Comp. 80	7-NH_2	N	-0.30	-1.79	-2.07	5.08	3.01		
Comp. 81	7-COOH	N	-0.09	-4.35	-4.43	9.12	4.69		

[a] Compounds are named as "series number-site number plus substitution group number" a: -F, b: -Cl, c: -Br, d: -NO_2, e: -CH_3, f: -CF_3, g: -OCH_3, h: -OH, i: -NH_2, j: -COOH.
[b] Substitution number and group.
[c] CH is "series 1" and N is "series 2".
[d] Energy changes in this Table are in kcal/mol.
[e] $\Delta\Delta G = \Delta G$(substituent) - ΔG(original).
[f] Inhibition constants are in *n*M.

Table S2 Fungus growth inhibitory rate of target compounds (200 mg/L).

Comp. No.	R	X	*Sphaerotheca fuliginea* (Schlecht.) Poll.	*Pseudoperonospora cubensis* (Berk. et Curt.)
Comp. 12	5-F	CH	99 %	90 %
Comp. 26	6-CH_3	CH	81 %	97 %
Comp. 18	5-OCH_3	CH	100 %	100 %
AZ			100 %	94 %

Table S3 Data collection and structure refinement statistics

PDB Accession code	4U3F
Space Group	P212121
Cell parameters	167.251 181.483 239.631 90 90 90
Resolution range	15 – 3.23
Number of Atoms Refined	32,939

Number of Reflections	11,3869
Redundancy	6.0
Completeness	98.3
Cryst. R Value	0.218
Free R Value	0.281
B Values	---
From Wilson Plot	59.4
Mean atomic B Value	56.9
ESD (Å) Phenix ML-based	0.47
RMS Dev. from Ideal:	---
Bond Lengths	0.010
Bond Angles	1.417
Dihedral Angles	18.2
Chirality Improper Angles	0.102

References:

1. Duan, Y. et al. A point-charge force field for molecular mechanics simulations of proteins based on condensed-phase quantum mechanical calculations. *J Comput Chem* **24**, 1999-2012 (2003).
2. Wang, J., Wolf, R.M., Caldwell, J.W., Kollman, P.A. & Case, D.A. Development and testing of a general amber force field. *J Comput Chem* **25**, 1157-1174 (2004).
3. Jakalian, A., Jack, D.B. & Bayly, C.I. Fast, efficient generation of high-quality atomic charges. AM1-BCC model: II. Parameterization and validation. *J Comput Chem* **23**, 1623-1641 (2002).
4. Jorgensen, W.L., Chandrasekhar, J., Madura, J.D., Impey, R.W. & Klein, M.L. Comparison of simple potential functions for simulating liquid water. *J Chem Phys* **79**, 926-935 (1983).
5. Darden, T., York, D. & Pedersen, L. Particle mesh Ewald: An N . log(N) method for Ewald sums in large systems. *J Chem Phys* **98**, 10089-10092 (1993).
6. Essmann, U., Perera, L. & Berkowitz, M.L. A smooth particle mesh Ewald method. *J Chem Phys* **103**, 8577-8593 (1995).
7. Ryckaert, J.P., Ciccotti, G. & Berendsen, H.J.C. Numerical integration of the Cartesian equations of mot ion of a system with constraints: molecular dynamics of n-alkanes. *J Comput Phys* **23**, 327-341 (1977).
8. Yu, L. & Yu, C.A. Quantitative Resolution of Succinate-Cytochrome-C-Reductase into Succinate-Ubiquinone and Ubiquinol-Cytochrome-C-Reductases. *J Biol Chem* **257**, 2016-2021 (1982).
9. Zhao, P.L. et al. Subnanomolar inhibitor of cytochrome bc1 complex designed by optimizing interaction with conformationally flexible residues. *J Am Chem Soc* **132**, 185-194 (2010).
10. Yu, C.A. & King, T.E. Preparation and Properties of Cardiac Cytochrome-C1. *J Biol Chem* **247**, 1012-1019 (1972).
11. Rieske, J.S. Preparation and properties of reduced coenzyme Q-cytochrome *c* reductase (complex III of the respiratory chain). *Methods Enzymol* **10**, 239-245 (1967).
12. Luo, C., Long, J.G. & Liu, J.K. An improved spectrophotometric method for a more specific and accurate assay of mitochondrial complex III activity. *Clin Chim Acta* **395**, 38-41 (2008).
13. Chretien, D. et al. Revisiting pitfalls, problems and tentative solutions for assaying mitochondrial respiratory chain complex III in human samples. *Curr Med Chem* **11**, 233-239 (2004).

14. Fisher, N., Bourges, I., Hill, P., Brasseur, G. & Meunier, B. Disruption of the interaction between the Rieske iron-sulfur protein and cytochrome b in the yeast bc1 complex owing to a human disease-associated mutation within cytochrome b. *Eur J Biochem* **271**, 1292-1298 (2004).
15. Fisher, N. et al. Modeling the Qo site of crop pathogens in Saccharomyces cerevisiae cytochrome b. *Eur J Biochem* **271**, 2264-2271 (2004).
16. Fisher, N. & Meunier, B. Re-examination of inhibitor resistance conferred by Q(o)-site mutations in cytochrome b using yeast as a model system. *Pest Manag Sci* **61**, 973-978 (2005).
17. Wang, B.-L. et al. Synthesis and Biological Activity of Some Novel Trifluoromethyl-Substituted 1,2,4-Triazole and Bis(1,2,4-Triazole) Mannich Bases Containing Piperazine Rings. *J Agric Food Chem* **58**, 5515-5522 (2010).
18. Wang, L., Li, B.J., Xiang, W.S., Shi, T.X. & Liu, C.L. Control Effects of Pyraoxystrobin on Cucumber Powdery Mildew. *Agrochemicals* **47**, 378-380 (2008).
19. Zhang, Z.Y., Dai, G.H., Zhuge, Y.Y. & Li, Y.B. Protective effect of Robinia pseudoacacia Linnl extracts against cucumber powdery mildew fungus, Sphaerotheca fuliginea. *Crop Prot* **27**, 920-925 (2008).
20. Otwinowski, Z. & Minor, W. Processing of X-ray diffraction data collected in oscillation mode. Vol. 276 307-326 (Elsevier, 1997).
21. Afonine, P.V. et al. Towards automated crystallographic structure refinement with phenix.refine. *Acta Crystallogr Sect D: Biol Crystallogr* **68**, 352-367 (2012).
22. Moriarty, N.W., Grosse-Kunstleve, R.W. & Adams, P.D. electronic Ligand Builder and Optimization Workbench (eLBOW): a tool for ligand coordinate and restraint generation. *Acta Crystallogr Sect D: Biol Crystallogr* **65**, 1074-1080 (2009).
23. Chen, V.B. et al. MolProbity: all-atom structure validation for macromolecular crystallography. *Acta Crystallogr Sect D: Biol Crystallogr* **66**, 12-21 (2010).
24. Jones, T.A., Zou, J.Y., Cowan, S.W. & Kjeldgaard, M. Improved methods for building protein models in electron density maps and the location of errors in these models. *Acta Crystallogr* **A47**, 110-119 (1991).
25. Kraulis, P.J. MOLSCRIPT: a program to produce both detailed and schematic plots of protein structures. *J Appl Cryst* **24**, 946-950 (1991).
26. Merritt, E.A. & Murphy, M.E.P. Raster3D version 2.0. A program for photorealistic molecular graphics. *Acta Crystallogr Sect D: Biol Crystallogr* **50**, 869-873 (1994).
